# Insights into Carbapenem Resistance in *Vibrio* Species: Current Status and Future Perspectives

**DOI:** 10.3390/ijms232012486

**Published:** 2022-10-18

**Authors:** Joanna Xuan Hui Goh, Loh Teng-Hern Tan, Jodi Woan-Fei Law, Kooi-Yeong Khaw, Nurul-Syakima Ab Mutalib, Ya-Wen He, Bey-Hing Goh, Kok-Gan Chan, Learn-Han Lee, Vengadesh Letchumanan

**Affiliations:** 1 Novel Bacteria and Drug Discovery Research Group (NBDD), Microbiome and Bioresource Research Strength (MBRS), Jeffrey Cheah School of Medicine and Health Sciences, Monash University Malaysia, Bandar Sunway, Selangor Darul Ehsan 47500, Malaysia; 2 Clinical School Johor Bahru, Jeffry Cheah School of Medicine and Health Sciences, Monash University Malaysia, Johor Bahru 80100, Malaysia; 3 Biofunctional Molecule Exploratory (BMEX) Research Group, School of Pharmacy, Monash University Malaysia, Bandar Sunway, Selangor Darul Ehsan 47500, Malaysia; 4 UKM Medical Molecular Biology Institute (UMBI), Universiti Kebangsaan Malaysia, Kuala Lumpur 56000, Malaysia; 5 Faculty of Health Sciences, Universiti Kebangsaan Malaysia, Kuala Lumpur 50300, Malaysia; 6 State Key Laboratory of Microbial Metabolism, Joint International Research Laboratory of Metabolic and Developmental Sciences, School of Life Sciences & Biotechnology, Shanghai Jiao Tong University, Shanghai 200240, China; 7 College of Pharmaceutical Sciences, Zhejiang University, Hangzhou 310058, China; 8 Division of Genetics and Molecular Biology, Institute of Biological Sciences, Faculty of Science, University of Malaya, Kuala Lumpur 50603, Malaysia; 9 International Genome Centre, Jiangsu University, Zhenjiang 212013, China

**Keywords:** antimicrobial, antibiotics, resistance, carbapenem, *Vibrio*, indicator strain, environmental, mechanism

## Abstract

The increasing prevalence of resistance in carbapenems is an escalating concern as carbapenems are reserved as last-line antibiotics. Although indiscriminate antibiotic usage is considered the primary cause for resistance development, increasing evidence revealed that inconsequential strains without any direct clinical relevance to carbapenem usage are harboring carbapenemase genes. This phenomenon indirectly implies that environmental microbial populations could be the ‘hidden vectors’ propelling carbapenem resistance. This work aims to explore the carbapenem-resistance profile of *Vibrio* species across diverse settings. This review then proceeds to identify the different factors contributing to the dissemination of the resistance traits and defines the transmission pathways of carbapenem resistance. Deciphering the mechanisms for carbapenem resistance acquisition could help design better prevention strategies to curb the progression of antimicrobial resistance development. To better understand this vast reservoir selecting for carbapenem resistance in non-clinical settings, *Vibrio* species is also prospected as one of the potential indicator strains for carbapenem resistance in the environment.

## 1. Introduction

The inception of antibiotics has revolutionized medicine in many ways. Antibiotics are invariably the primary recourse when confronting bacterial infections. Nonetheless, the progressive antimicrobial resistance (AMR) development is gradually rendering antibiotics ineffective against pathogens and slowly jeopardizing the healthcare system. To date, AMR is one of the most pressing health concerns globally, leading to increasingly complex treatment regimes, prolonged hospitalizations, higher morbidity and mortality rates, and accounting for massive social and economic burdens [1,2]. This predicament is further aggravated by the emergence of multi-drug-resistant superbugs that are resistant to carbapenems [3,4,5].

The consequences are frightening, as the World Health Organization [6] has classified carbapenems as ‘critically important antimicrobials’ for two reasons. Firstly, this class of antimicrobial constitutes one of the limited options available to treat multi-drug resistant Enterobacteriaceae, including *Klebsiella pneumoniae*, *Escherichia coli,* and *Enterobacter* sp. [3,6]. Secondly, carbapenems are used to treat infections caused by bacteria transmitted from non-human sources such as *Salmonella* sp. and *E. coli* [6]. This implies carbapenem resistance may silently disseminate on several fronts beyond the clinical context, therefore being formidable to control. Cumulating reports revealed that local and regional outbreaks of carbapenem-resistant *K. pneumoniae* infections are difficult to contain and are expanding globally [7,8,9,10,11]. Moreover, carbapenem resistance culminates in skyrocketing clinical and economic costs. A retrospective study concluded that contracting carbapenem-resistant *Acinetobacter baumannii* infection significantly elevated the total medical cost by 1.5-fold compared to the carbapenem-susceptible bacterial infection [12]. Furthermore, metanalyses revealed that dealing with carbapenem-resistant infectious agents increases the patient’s mortality rate by two to three-fold [5,13].

Despite being considered a clinical issue, surveillance has demonstrated that resistant strains are no longer confined to clinical settings. It is indeed alarming when the prevalence of carbapenem-resistant organisms has propagated to a myriad of ecospheres, including aquatic environments such as wastewater treatment plants [14,15], drinking water [16], river [17,18], urban lake [19], estuary [20], and coastal water [21,22,23]. In addition to that, resistant strains are also identified in diverse food sources, including food-producing animals [24], fresh vegetables [25], frozen meat [26], and raw foods, in particular seafood samples [27,28,29,30,31,32], thus signaling a direct threat to human health.

Interestingly, there is evidence supporting the persistence of antibiotic resistance in human pathogens isolated from environmental samples without a direct clinical reservoir [33]. In comparison, another study reported the high similarity between resistant strains isolated from environmental settings and clinical isolates using Enterobacterial repetitive intergenic consensus sequence polymerase chain reaction (ERIC-PCR) and pulsed-field gel electrophoresis (PFGE) [34]. Xin, Zhang, Wu, Zhang, and Niu [23] further confirmed environmental bacteria’s crucial role in carrying the carbapenemase genes. Ignoring this critical observation would invariably overlook a potential missing link in defining the carbapenem resistance pathways. The natural environment that harbors a rich microbial diversity is a reservoir propelling AMR development [35]. However, the risk contributed by the environmental reservoir remains speculative due to the lack of surveillance. Moreover, the mechanisms for resistance development are largely understudied, thereby leaving several knowledge gaps to prevail.

In this regard, this review seeks to gather primary works concerning the antimicrobial susceptibility profiles of *Vibrio* spp., particularly those demonstrating resistance to carbapenems, to assess the prevalence and susceptibility patterns in the environment in different parts of the globe. It is alarming to note that carbapenem-resistant *Vibrio* isolates have been identified in all continents. This suggests a global crisis rather than an isolated phenomenon. The surveillance report condensed in this review helps illustrate the extent and severity of the issue.

This review aims to explore the carbapenem-resistance profile of *Vibrio* spp. across diverse settings. In addition to that, this review also compiles relevant information from the available literature to decipher the mechanisms driving AMR to carbapenems, including those at molecular levels. It is hoped that by identifying the pathways of antimicrobial resistance dissemination and acquisition from an environmental standpoint, this review will shed light on the evolution and dynamics of carbapenem resistance development. To better understand this vast reservoir selecting for carbapenem resistance in non-clinical settings, *Vibrio* species is also prospected as one of the potential indicator strains for carbapenem resistance in the environment. Addressing this knowledge gap will certainly provide an added edge in the effort to combat this global crisis and facilitate the implementation of pragmatic strategies to nip the AMR issues in the bud and promote public safety.

## 2. Carbapenems

**Figure 1:**
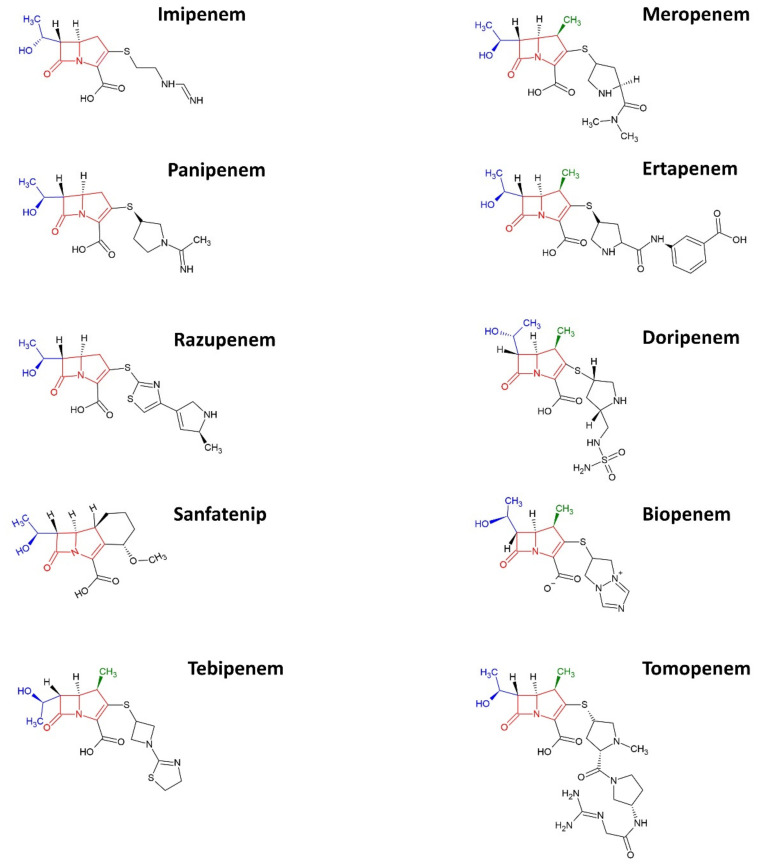
Molecular structures of the conventional and newer antimicrobials in the carbapenem class: Imipenem, Panipenem, Meropenem, Ertapenem, Doripenem, Biapenem, Razupenem, Sanfatenip, Tebipenem, and Tomopenem. Red: carbapenem structure; blue: moiety that confers steric hindrance towards the action of β-lactamases; green: 1-ß methyl group that confers resistance against DHP-1 hydrolysis.

Carbapenems are characterized by the distinctive carbapenem structure (see red structure in Figure 1) that comprises the β-lactam ring. This unique combination in their molecular structure makes this group of antibiotics exceptionally stable when confronted by β-lactamases [36], mediated by either the TEM, SHV, CTX, or OXA genes [37]. Due to the steric hindrance conferred by the 6-α-1R-hydroxyethyl moiety (see blue structure in Figure 1) at the β-lactamase binding site [38,39], carbapenems are significantly more stable and more effective than other β-lactams such as penicillin and cephalosporins [40,41]. Therefore, carbapenems have become the drug of choice when treating the strains producing the extended-spectrum β-lactamases (ESBLs), which are resistant to other second and third-generation β-lactams [36]. Nevertheless, carbapenems are often reserved as the “last resort” or “last-line agents” for multi-drug resistant bacteria [42].

Most importantly, carbapenem is the antimicrobial class that covers the broadest spectrum of bacteria amongst all other β-lactams [36]. Carbapenems are broad-spectrum antimicrobials effective against a diverse range of Gram-positive and Gram-negative, aerobic and anaerobic bacteria, with only several known exceptions. For example, methicillin-resistant *Staphylococcus aureus* (MRSA) and ampicillin-resistant *Enterococcus faecium* are intrinsically resistant to carbapenems, likely due to the poor affinity with PBPs in these cells [40,43,44,45]. *Stenotromonas maltophilia* is also resistant to carbapenems, attributed to the distinct metallo-ß-lactamases that are very effective at hydrolyzing carbapenems [40,46]. Furthermore, carbapenems are relatively safer and present with fewer adverse effects when compared to other last-line agents such as fosfomycin and polymyxin B, which are known for their toxicity concerns [47]. Nevertheless, carbapenems are still associated with side effects such as skin rashes, infusion-site complications, gastrointestinal distress, and anaphylaxis, although they are generally well tolerated [41,48].

Mechanism-wise, carbapenems exhibit bactericidal activity. The relatively smaller molecular sizes of carbapenems significantly contributed to the activity enhancement. Moreover, their existence in the form of zwitterions also facilitates substantial penetration through the bacterial cell wall [44]. After entering the bacteria through the outer membrane proteins, known as porins, carbapenem traverses through the periplasmic space and acylate the penicillin-binding proteins (PBPs) such as the carboxypeptidases, transglycosidases, and transpeptidases [42,49]. Most carbapenems demonstrate a high affinity with the essential PBPs in a diverse spectrum of bacteria [44,50]. Inhibition of these critical enzymes involved in peptidoglycan formation hampers the proper bacterial cell wall synthesis. Therefore, the treated bacteria undergo lysis under osmotic pressure without a sturdy cell wall [42].

In 1985, imipenem was introduced as the first carbapenem. Although renowned for its exceptional ability to withstand β-lactamase hydrolysis and high PBP affinity, a major drawback of imipenem is its susceptibility to dehydropeptidase I (DHP-1) deactivation. Therefore, imipenem must be co-administered with cilastatin, a DHP-1 inhibitor. Following that, advances in the pharmaceutical field brought in other carbapenems such as biapenems, doripenem, ertapenem, and meropenem. These carbapenems that have been introduced subsequently demonstrated increased stability to withstand DHP-1 hydrolysis due to the addition of a methyl group (CH_3_—) at the 1-ß position (see green structure in Figure 1) except panipenem, which necessitates the co-administration of DHP-1 inhibitor, betamipron [38,41,42].

Although categorized under the same class, the carbapenems have a slightly different range of activity. In this regard, El-Gamal, Brahim, Hisham, Aladdin, Mohammed, and Bahaaeldin [38] proposed a three-group classification scheme for carbapenems according to their range of activity. Imipenem is comparatively more efficacious against Gram-positive strains than other carbapenems, particularly against *Pseudomonas aeruginosa* [38]. While maintaining the bactericidal efficacy against *P. aeruginosa,* adopting the pyrrolidine ring at the C-2 position significantly diminished meropenem’s renal and central toxicity effects [38]. Meropenem is comparatively more potent than imipenem in inhibiting Gram-negative isolates, although its action against Gram-positive isolates is lesser than imipenem. Meropenem was reported to be two-fold more potent than ciprofloxacin against *Vibrio parahaemolyticus*, and four to 16-fold more effective than imipenem against *Vibrio cholerae* [51]. However, a sharp decline in sensitivity towards *Acinetobacter sp.* has been reported for meropenem over the years [52]. Due to its unique meta-substituted benzoic acid side chain, ertapenem has a longer half-life and is preferred for its once-daily dosing regimen. However, ertapenem possesses limited efficacy against non-fermenting Gram-negative isolates [38,41]. Biapenem and doripenem have comparable activity to imipenem in targeting Gram-positive bacteria. These antibiotics effectively treat various nosocomial infections and a wide range of Gram-negative strains, including the ESBL-producing isolates. To illustrate, doripenem is highly effective towards *A. baumannii*, *E. coli*, *K. pneumoniae,* and *P. aeruginosa* [38,41,53,54]. Additionally, doripenem has higher tolerability and a lower tendency to trigger seizures. Panipenem, on the contrary, is inactive against *P. aeruginosa*, although its activity against other Gram-negative aerobic bacteria is on par with imipenem and is two to four-fold more efficacious than meropenem [38,41]. Each of these carbapenems has its particular advantages and drawbacks. Nevertheless, only a brief description is given in this review as the detailed pharmacokinetics and pharmacodynamics report have been covered extensively in previous reviews [38,40,41,42].

In recent years, carbapenem resistance has been noted due to the widespread use of carbapenems. The prevalence of inconsequential Gram-negative rods in the environment and clinically relevant Gram-negative strains such as *A. baumannii*, *P. aeruginosa* and Enterobacterales resistant to carbapenems continues to rise at an alarming rate [55,56]. This phenomenon is one of the imminent threats to public health because these mobile resistance elements could be easily transmitted between non-human sources, thus making the outbreak challenging to contain epidemiologically [6]. The acquired carbapenemase traits not only conferred resistance to carbapenems, but also made the strain practically resistant to other ß-lactams and multiple antibiotics of different classes [21,57,58,59]. For instance, all *V. vulnificus* strains carrying the New Delhi metallo-ß-lactamase (*bla*_NDM-1_) (gene that encodes carbapenemase which make the bacteria resistant to carbapenems) sampled from the recreational beaches in Nigeria were found to be resistant to at least 17 antibiotics, with the highest record of 24 types [60]. Moreover, infections induced by carbapenem-resistant Enterobacteriaceae are challenging to treat and associated with increased mortality rates due to the minimal treatment options available [61].

At present, there are only a few potential replacement carbapenems in the pipeline [38,42]. Although newer carbapenems such as razupenem, sanfetrinem, tebipenem, and tomopenem have been introduced, there is a need to seek a sustainable solution to remedy the AMR scenario. In contrast to all conventional carbapenems that must be administered via the parenteral route, tebipenem is the first oral carbapenem under development. It is introduced as a prodrug in ester form to facilitate intestinal absorption for higher bioavailability [38,41,42]. Another fruit of contemporary research is the emergence of trinem, also known as tribactam, a new class of ß-lactam represented by the sanfetrinem. Sanfetrinem has the carbapenem backbone and is also meant to be orally administered and target intracellular pathogens [38,42]. Tomopenem is another novel carbapenem that is efficacious at overcoming the intrinsic resistance of carbapenems towards MRSA due to the PBP affinity modulatory effect arising from the unique side chains adopted [38,42]. However, many of these leads only progressed to the clinical phase before being discontinued [42]. For instance, razupenem, the potential agent for polymicrobial anaerobic infection, was withdrawn at the early clinical trial due to the high rate of adverse events [38]. In this sense, it is necessary to identify the pathways and vectors propelling carbapenem resistance to break the chain of transmission.

## 3. *Vibrio* spp.

To better appreciate *Vibrio* spp. as the central focus of this review, it is fitting to deliberate on several noteworthy features of the genus to provide context. Members of the Vibrionaceae family are highly diverse. More than 142 species from the genus *Vibrio* have been discovered [62]. However, its taxonomy and phylogeny have continuously been revised with the progressive identification of new species [63,64,65]. Among the many strains, *V. cholerae* is one of the most notorious pathogens for causing cholera, gastrointestinal and extraintestinal infection, bacteremia, and even death [66,67,68,69,70]. Likewise, *V. vulnificus* is a classical opportunistic pathogen recognized as the leading cause of mortality among *Vibrio*-associated infections. This infection transmitted through the exposure of open wounds to water bodies containing the pathogens or consumption of contaminated seafood often results in septicemia and necrotizing fasciitis [21,71,72,73]. *V. parahaemolyticus* is one of the common agents for food-borne infections, primarily spreading through undercooked seafood consumption. However, this infectious agent typically results in acute gastroenteritis manifesting symptoms such as abdominal cramps, diarrhea, nausea, headache, chills, and fever, and is usually not life-threatening [74,75]. Furthermore, *V. alginolyticus, V. carchariae, V. cincinnatiensis, V. damsela, V. fluvialis, V. furnissi, V. hollisae, V. metschnikovii,* and *V. mimicus* are also examples of clinically important species that have been associated with diarrhea [70,76,77,78]. Several species such as *V. parahaemolyticus, V. harveyi, V. alginolyticus, V. anguillarum, V. owensii*, *V. campbellii,* and *V. mediterranei* are infectious to cultured aquatic livestock such as shrimps, fishes, oysters, and mussels [20,79,80]. This review will focus more on the economically and clinically relevant strains such as *V. cholerae, V. parahaemolyticus,* and *V. vulnificus*. However, other species such as *V. aestuarianus, V. agarivorans, V. antiquaries,* *V. brasiliensis, V. campebelli, V. chagasii, V. coralliilyticus, V. cyclitrophicus, V. damsela, V. diabolicus, V. fortis, V. fujianensis, V. furnissii,* *V. gigantis, V. hangzhouensis,* *V. ichthyoenteri, V. navarrensis, V. pelagius, V. pomeroyi, V. rotiferanius, V. scophthalmi, V. shilonii, V. sinalooensis, V. tasmaniensis, V.tubiashii,* and *V. xuii* have been identified along the screening process [20,81,82,83,84,85]. It should be noted that not all *Vibrio* spp. are pathogenic.

In general, *Vibrio* spp. are facultatively anaerobic, Gram-negative bacteria that appear as curved or straight rods with a single polar flagellum for locomotion. They are mainly halophilic and oxidase-positive isolates [21,86]. *Vibrio* spp. can thrive in many aquatic habitats, including freshwater, estuarine, and marine ecosystems [71,77]. Some species are also considered commensal organisms and play essential roles in nutrient cycling within the natural aquatic ecosystems. Interestingly, the variability and diversity of the *Vibrio* population are due to geographical dynamics, salinity variation, climatic, and seasonal influences [87,88,89,90]. Typically, *Vibrio* spp. infection cases increase markedly during warmer seasons [20,33].

### 3.1. Vibrio spp. as the Indicator Strain of Carbapenem Resistance in the Environment

Several intrinsic and extrinsic characteristics of *Vibrio* spp. made it one of the prospectus indicators strains for carbapenem resistance in the environment. Firstly, the high genomic plasticity of *V. cholerae* featured in recent reviews [59,91] further reaffirmed our selection. The genetic makeup of the genus makes them remarkably competent to adapt to hostile environments and resist the action of antimicrobial agents [92]. Like the carbapenem resistant *A. baumannii* and *P. aeruginosa, Vibrio* spp. are also proven capable of assuming the spherical form and entering into dormancy under hostile conditions [93]. They can exist as persister cells as part of their antimicrobial resistance potential (see Section 5.4). Furthermore, the bacterium can competently acquire environmental DNA through horizontal gene transfer (HGT) [59] (see Section 5.3). Moreover, this natural competency is further enhanced in the presence of chitin, an abundant biopolymer in the aquatic environment [94,95]. Inspection of the genomic profile of four extensively drug-resistant and multidrug-resistant *V. cholerae* strains revealed the close association of antibiotic resistance genes with mobile genetic elements (MGEs) [59].

The remarkable genetic plasticity in *Vibrio* spp. is hypothesized to be conferred by two sophisticated types of machinery, namely, the bi-chromosomal genome and the superintegron, which act as an apparatus to capture genes in the chromosome and to facilitate the adaptive function under antibiotic selective pressure [91]. Recombination of the genetic cassette into superintegron enables the continuous recruitment of exogenous genetic elements that confer adaptive traits. This genetic system provides a low-cost means to stockpile adaptive functions as the gene cassettes acquired can be reshuffled, silenced, or recovered within a tightly regulated system along the innovative process of evolution [91]. A bipartite genomic architecture provided an evolutionary advantage for Vibrionaceae as the distribution of genes critical for growth and survival on the larger chromosome offered exceptional stability in conserving the essential traits. In contrast, the smaller chromosome demonstrates higher plasticity and evolutionary rate. It ably amasses innovative genes to support adaptation [91,96,97]. Building on the literature, a third plausible contributing factor to genomic plasticity would be the presence of the multicopy plasmids in *Vibrio* spp. Plasmid profiling revealed that 173 *V. parahaemolyticus* amongst the 200 strains isolated from shellfish samples (86.5%) carry at least one plasmid. Meanwhile, some strains contain up to seven plasmids with varying DNA band sizes [29]. San Millan et al. [98] successfully demonstrated the role of multicopy plasmids in augmenting the phenotypic expression of antibiotic resistance. Results showed that multicopy plasmids mediated a remarkable 128-fold increase in ceftazidime resistance which is in stark contrast to the modest two-fold increase in the similar mutation induced in the chromosomal copy. Stemming from this, the plasmid-mediated carbapenem resistance in *Vibrio* spp. awaits further vindication.

Apart from that, due to their adaptive abilities and metabolic diversity, members of this Vibrionaceae family can ubiquitously inhabit diverse aquatic niches globally [90,99,100]. Interestingly, *Vibrio* spp. demonstrates extraordinary fitness and can ably adjust to accommodate the changing environment [92]. On top of that, *Vibrio* spp. can exist in a multitude of organisms, ranging from amoeba [101], yeast [102], insects [103], planktons [104], crustaceans [80], to infected humans [105,106]. The persistence of *Vibrio* spp. in both abiotic and biotic environments increase their capacity to act as the vector for AMR, thus bridging the transmission from the external environment to human beings (see Section 6). *Vibrio* spp. sustains a unique relationship with humans as it is a common pathogen afflicting humans. To illustrate, *Vibrio* spp. such as *V. cholerae,* can be easily transmitted to another individual through the fecal–oral route. In the diseased state, the proximity and mingling of these *Vibrio* strains pose a risk of transferring the resistant genes to the gut microbiota [107]. The existence of virulence genes within the strains and the ability to form biofilm further enhances the infectivity, survival, and transmission rate of the pathogen, thus lending impetus to AMR development and increasing the hazards and risk to human health [75]. This distinctive trait can create the opportunity to align microbiological data to environmental data, which would be another valuable piece of information to gauge the risks to human health [20].

Besides, *Vibrio* spp. are strains of high public health importance. Most importantly, this genus’s isolation and characterization work are relatively well established. Various rapid and cost-effective isolation techniques specific to *Vibrio* spp. have been introduced to facilitate rapid identification of the bacteria, even up to the species level [108,109,110,111,112,113,114]. The clinical signs vary greatly depending on the infectious species and serotypes [71]. While some *Vibrio* infections can be self-limiting, antibiotics are often part of the treatment protocol for critical *Vibrio* infections [21,115]. The increasing multi-drug resistance (MDR) trend observed in *Vibrio* spp. is a cause of increasing concern as this phenomenon will further limit the already scanty therapeutic options available [33,73,116,117,118,119,120,121]. To illustrate, Sperling et al. [122] reported that MDR was observed amongst 76% of the *V. parahaemolyticus* samples isolated in shrimps in Ecuador. At the same time, the *V. alginolyticus* strains were also found resistant to up to 18 antibiotics. Similarly, high frequency of resistance (>50%) of the *V. parahaemolyticus* isolated from freshwater and marine fishes in Malaysia has been noted against ampicillin, amikacin, kanamycin, as well as the third-generation cephalosporins such as cefotaxime [27]. These findings collaborate with the results of Tan et al. [123] and Sadat, El-Sherbiny, Zakaria, Ramadan, and Awad [75]. In addition, the significantly high multiple antimicrobial resistance (MAR) index was noted in many *Vibrio* spp. isolated from diverse samples, suggesting an imminent threat [20,27,34,75,123]. The emergence of *Vibrio* spp. as a notorious MDR pathogen will engender additional hurdles in disease management. Alarmingly, a recent large-scale analysis on the resistance profiles of *Vibrio* spp. indicated that no single antibiotic is universally effective against all cases of *Vibrio*-related infections due to the heterogeneous resistance pattern developed across the species, which includes the resistance to the last-line agents such as carbapenems [124]. Therefore, surveillance for resistance against last-resort antibiotics such as carbapenem is necessary to expedite the implementation of appropriate preventive strategies at early phases of AMR development.

Last but not least, the *Vibrio* spp. are selected as the indicator strains for novelty reasons. *Vibrio* spp. are typically given less attention when it comes to studying carbapenem resistance as compared to the primary clinically relevant strains such as *A. baumannii* [125,126] and specific strains of the Enterobacteriaceae family [127,128] such as *K. pneumoniae* [7,129]. Although using carbapenem to treat *Vibrio*-induced infection is uncommon, one cannot neglect the possibility of *Vibrio* spp. harboring the carbapenem resistance trait. Stemming from the line of reasoning and preliminary data gathered, the *Vibrio* species is postulated as one of the indicator strains for assessing the severity of carbapenem-resistance among the environmental isolates. Turning a blind eye to this critical observation would possibly forego a potential missing link in defining the carbapenem resistance pathways.

## 4. Prevalence of Carbapenem Resistance

Due to its subordinate clinical importance, the epidemiology of the carbapenem-resistant *Vibrio* spp. is poorly established. However, it is crucial to understand the extensiveness of carbapenem resistance among *Vibrio* isolates early. Once acquired, the resistance genes may disseminate to other bacteria and rapidly amplify the development of carbapenem resistance. Identifying the reservoir of carbapenem resistance at an earlier phase will buy time to strategize appropriate measures to prevent other bacterial species from enduring the untoward fate of carbapenem-resistant Enterobacteriaceae (CRE). In this regard, surveillance beyond the clinical setting is necessary, particularly for *Vibrio* spp., which could be a significant environmental reservoir.

In this realization, antimicrobial surveillance of *Vibrio* species in non-clinical settings has been gradually increasing in recent years. In line with the hypothesis, carbapenem-resistant *Vibrio* spp. has been identified beyond the clinical territories. Antibiogram signatures revealed traits of resistance among *Vibrio* isolates from recreational beaches [60], coastal water [130], river water [131], and urban tropical estuary [20]. Additionally, surveillance results testified to the presence of carbapenem-resistant isolates in food such as salad [132] and seafood, particularly shrimps, mussels, and marine and freshwater fishes [27,28,29,30,31,34,75,81,83,121,133,134]. However, information is lacking concerning the relationship between these *Vibrio* strains isolated at different sites. The distance between sampling sites, sampling time, sampling method, and confounding factors may indirectly affect the surveillance outcome. Furthermore, the result is also subjected to environmental conditions such as temperature, salinity, and ion contents when conducting the antimicrobial susceptibility test (AST).

There may be slight discrepancies between the outcomes due to the complexity between genetic expression and phenotype. For example, despite the detection of the *V. alginolyticus* metallo-β-lactamase (VAM-1) gene, isolate Vb1579 did not demonstrate resistance to imipenem (0.06 mg/L) and meropenem (0.12 mg/L) in AST [135]. It is important to note that strains harboring carbapenemase genes but portrayed as susceptible under standard AST tests are often excluded in some prevalence studies [58]. This is of concern as there is a risk of disseminating the carbapenem resistance genes in these ‘asymptomatic carriers’ to other bacteria species through HGT. To illustrate, the VAM-containing plasmid isolated from the carbapenem susceptible strain when transformed into *E. coli* H5α, conferred the recipient strain a high resistance level to imipenem, meropenem, and ertapenem [135].

Similarly, the *bla*_NDM-1_ gene from an apparent imipenem-susceptible *V. parahaemolyticus* was successfully conjugated to *E. coli* UB5201 [74]. The concept of “proto” or “silent” resistance genes, which refer to genes without resistance expressing capability, has been detailed by Perry et al. [136]. Martínez et al. [137] examined the risk of such antimicrobial resistance gene (ARG) transference to pathogens and proposed a method to quantitate the risk. Nonetheless, these different approaches should be viewed as complementary rather than contradictory, as the results offered a multi-dimensional perspective on carbapenem resistance within the bacteria. The fragmentary AST results of carbapenem-resistance for *Vibrio* spp. captured from worldwide surveillance and the respective carbapenemase phenotypic and genotypic profiles have been tabulated in Table 1.

In summary, carbapenem-resistant *Vibrio* isolates have been identified in all continents (see Table 1) except Antarctica. Reassuringly, a majority of the degrees of resistance are not high, mostly less than 10%, except for surveillance reports for environmental samples from Chhatak, Bangladesh [139], Lagos, Nigeria, Uganda [159], Eastern Cape, South Africa [131], Norway [83]; clinical samples in India [57,59]; seafood samples in Korea [134], Malaysia [27,32], Nigeria [121], and Egypt [75]. The antibiotic susceptibility pattern associated with the 16 *Vibrio* species covered in the existing surveillance report is highly diverse, with the carbapenem-resistance remarkably prevalent in *V. parahaemolyticus* [32,75,134], *V. cholerae* [59,140], *V. vulnificus* [60], *V. fluvialis* [57,131], *V. anguillarum* [83], and *V. alginolyticus* [75]. More worryingly, an increasing trend of intermediate-resistance transpires, indicating an evolution towards the expression of resistant phenotypes [140]. Take, for an example, the environmental samples (wastewater effluent) at Eastern Cape collected by Okoh and Igbinosa [158] in 2010, which presented with absolute susceptibility to carbapenem, whereas, after a decade, the environmental sample (river water) gathered by Gxalo, Digban, Igere, Olapade, Okoh, and Nwodo [131] at Eastern Cape demonstrated high levels of resistance to MER and IMI. The carbapenem-resistance among *Vibrio* isolates should garner increasing attention as their existence is widespread across the globe, spanning across diverse climate zones.

Although *Vibrio* surveillance is routinely conducted, few studies investigated the magnitude of carbapenem resistance among the *Vibrio* spp. due to its current lower level of clinical importance. Moreover, the studies are often conducted at arbitrary sites or frequently only focused on a few specific areas in the nation. The sporadic data hardly reflect the spatial distribution of the stable resistant strains across the country. Nevertheless, the information condensed in this review demonstrated the emerging trend of carbapenem resistance among *Vibrio* isolates across the globe. Notably, the prevalence of carbapenem-resistant traits amongst *Vibrio* spp., in reality, could be much higher than portrayed, given the under-represented samples.

## 5. Mechanism to Carbapenem Resistance Development

With the establishment of *Vibrio* as an emerging genus harboring carbapenem resistance, it is essential to identify how the species override the harmful effects of the antibiotics. Several antimicrobial resistance traits have been identified in pathogenic strains [92,116,167,168]. However, the pathways driving this resistance in *Vibrio* spp. are relatively understudied. Therefore, this section comprehensively summarizes the various mechanisms modulating carbapenem resistance in general. Several means conferring the carbapenem resistance have been identified, including reduced drug permeability, enhanced drug efflux, production of hydrolytic enzymes that inactivate carbapenem, dispersal of the resistance traits through HGT, and development of carbapenem tolerance [116,168,169].

### 5.1. Limiting the Intracellular Concentration of Drug

The outer membrane of bacteria, including the *Vibrio* spp., forms a selective permeability barrier against high molecular weight substances. Antibiotics such as carbapenem gain accessibility into the cell through outer membrane porins (OMPs), protein channels that facilitate the entry of solutes across the cell membrane. Suppression of OMP expression or alteration of its structural function can significantly diminish the fluidity of the membrane barrier, and therefore lower the permeability towards carbapenem and subsequently reduce the intracellular carbapenem concentration [42,92]. Achieving an optimal intracellular concentration of carbapenem is essential for antagonizing the intracellular target to ensure efficacy. Additionally, carbapenem MIC could be further elevated when coupled with the active drug extrusion effect generated by efflux pump protein which constitutes part of the tripartite protein complex. According to the sequence similarity, the efflux system can be broadly classified into five distinct families [42,47,168]. More extensive characterization work has been conducted for other species, such as *P. aeruginosa* [170,171] and *E. coli* [172,173]. Still, information is relatively sparse for *Vibrio* spp. Zago et al. [174] detected several efflux pump families for *Vibrio* spp., but none are carbapenem specific. Genomic analysis of *V. vulnificus* revealed the genetic traits coding for permeases and active drug transporters [33].

### 5.2. Carbapenemase

The predominant mechanism underpinning carbapenem resistance is the production of periplasmic enzymes that degrade carbapenems before reaching the PBP target. Although carbapenems are resistant to most β-lactamases, they are still subjected to the inactivation of a unique subset of enzymes, collectively known as carbapenemases. Worryingly, carbapenemase enzyme significantly increases the MIC, is frequently resilient to the β-lactamase inhibitors marketed, and demonstrates a versatile hydrolytic capacity against a broad spectrum of other enzymes, including almost all β-lactams. Thus, culminating in limited drug options available for treatment [36,47,167,175].

Besides the typical methods to investigate strains’ phenotypic and genotypic characteristics (see Section 4), the higher MIC value of ertapenem may offer good preliminary screening criteria for carbapenemase producers. However, additional tests are necessary for accurate confirmation [176,177]. Interestingly, the analysis revealed persistent β-lactamase expression and sustained functional activity regardless of the presence of an antimicrobial agent. This finding may imply the involvement of β-lactamase in other physiological functions apart from AMR [59]. However, this inference is yet to be verified by further research. Although carbapenemase secretion is one key contributor to carbapenem resistance development in *Vibrio* spp., resistance could arise from other mechanisms (see Section 5.1 and Section 5.4). Therefore, it should be noted that the phenotypic assessment for carbapenemase should not be the sole determinant for prevalence assessment, nor should the genotypic profiling be neglected as the reason for the poor expression of the genes remains elusive. Still, the risk of gene transference from non-fermenters to other bacteria was evident (see Section 5.3). State-of-the-art research identified several regulons such as VarR, VarG, and VArABCDEF in *V. cholerae,* which could be putative regulators to encode transcriptional activators, MBLs, and efflux pumps, respectively [107].

Carbapenemases comprise a wide variety of enzymes. The specification of each carbapenemase variant has been detailed in a previous review [178]. Each carbapenemase type is distinct in terms of amino acid identity, hydrolytic efficiency, carbapenem-resistant profile, epidemiology, and substrate preference. They can be classified based on the molecular (Ambler classification scheme) or functional aspects (Bush classification) [167,178,179]. Based on the functional classification, the hydrolytic enzymes for carbapenem are classified in 2df, 2f, 3a and 3b groups [167,180,181]. According to the more commonly adopted Ambler classification scheme, carbapenemases are categorized into three of the four Ambler classes: A, B, and D (see Table 2) [167,182].

Ambler class A, C, and D have a serine residue at the active site. The serine-β-lactamase uses serine as the nucleophile and catalyzes the hydrolytic effect on the β-lactam ring through an acyl-enzyme intermediate [31,42]. On the contrary, class B enzymes have distinctive zinc ion(s) serving as cofactor(s) to promote direct hydrolysis of the β-lactam ring through coordinating the polarized water ions for the oxy-anionic attack at the target site, hence the name metallo-β-lactamase (MBL) [149,184]. A comprehensive description of the molecular mechanisms has been detailed by Papp-Wallace, Endimiani, Taracila, and Bonomo [42] and Lu, Hsu, and Lin [73]. Besides the structural uniqueness, Ambler class B is implicated as the most clinically prevalent, versatile, and molecularly diverse group. MBLs can be subdivided into three subclasses, B1, B2, and B3, according to the structural feature and amino acid sequence [42,147]. Subgroup B2 demonstrates remarkable carbapenemase activity but is comparatively weaker against cephalosporins and penicillins, whereas MBL B1 and B3 have a relatively broad-spectrum activity to β-lactams including carbapenems [73,185]. Class C enzymes are not regarded as robust carbapenemases due to their limited capacity to hydrolyze carbapenem. However, carbapenem resistance may still be possible if overly expressed and coupled with other mechanisms such as reduced membrane permeability and augmented efflux pump expression [42,62]. Class D enzymes are also called the OXA enzyme or the oxacillinases attributed to the high hydrolytic capacity towards oxacillin. It is a heterogeneous group comprising members with narrow-spectrum β-lactamase as well as carbapenemase activity [3].

Although various carbapenemase types have been extensively identified in other Gram-negative species [58,167,186,187], carbapenemase producers are yet to be systematically characterized amongst *Vibrio* spp. Most of the reports available are sporadic and largely observational; the distribution and diversity of carbapenemase produced by *Vibrio* spp. documented could only be the tip of the iceberg. Nevertheless, those identified carbapenemase producers and the silent carriers of the carbapenemase genes of the *Vibrio* genus have been condensed in Table 1. Despite being inadequate, the comprehensive summary provides glimpses of the worldwide epidemiology of carbapenemase dissemination from various sample sources. To date, only ten types of carbapenemase have been identified in *Vibrio* spp. globally, with NDM-1 as the most prevalent type, followed by OXA, VIM, VCC, IMP, GES, VMB, VAM, and KPC; among which VCC-1 [31], Vmh [73], VAM-1 [135], VMB-1 [147], and VMB-2 [148] are novel types identified from *Vibrio* isolates.

Despite being recognized as the predominant and most widespread carbapenemase in other species such as *K. pneumoniae* and *P. aeruginosa* [47,175], only one study demonstrated the presence of *bla*_KPC_ genes in 6% of the total *Vibrio* spp. isolated from a river sample in South Africa [131]. Although first discovered just recently in 2008 in Sweden from the urinary *K. pneumoniae* and gastrointestinal *E. coli* from an Indian patient transferred from a hospital in New Delhi [188], NDM has instead eclipsed KPC and is identified now as the most prevalently discovered among the *Vibrio* spp. *bla*_NDM-1_ being the most common out of the 19 other variants [80], is found to be widely disseminated in *Vibrio* spp. across different countries including China [145], India [59,149,150,151], Vietnam [155], Nigeria [60], South Africa [157] and the United Kingdom [165]. The gene is either carried by the plasmid [57,149] (see Section 5.3) or integrated into the chromosome of *Vibrio* spp. [59]. In silico screening revealed that NMD-1 possesses higher catalytic efficiency and a more excellent drug profile than VIM-2 and IMP-1 attributed to the larger pocket opening and shorter distance between the Zn-I ion and the lactam-linked oxygen in the carbapenem structure [189].

VIM is another carbapenemase with the most similar amino acid homology (32%) to NMD. VIM-bearing *Vibrio* isolates have been identified in diverse sources, including seafood [145], wildlife [163], and environmental water samples [131,157]. Based on the limited information available, IMP and GES have only been detected in *Vibrio* spp. in recent years, and they seem to be endemic to South Africa [131,157]. VCC-1 demonstrates comparable kinetic parameters to IMI-1 and confers remarkable resistance to carbapenem, with a high catalytic turnover rate (*k_cat_*) for imipenem [31]. To date, VCC-1 has only been found in *V. cholerae* but not in other species [31,130]. A recent molecular survey projected a higher prevalence of the *bla*_VCC-1_ carriers in the environmental reservoir [130]. Interestingly, the *Vibrio* isolates harboring *bla*_VCC-1_ identified in Canada and Germany display different flanking genetic sequences, suggesting the gene was acquired on at least two occasions. However, the progenitor remains unknown [130].

Although OXA-carrying *Vibrio* spp. are still localized in South Africa [131] and Asian countries such as China [23], India [57], and Korea [133], an increasing trend in prevalence portends a cause of concern.VAM-1, VMB-1, and VMB-2 are novel MBLs recently derived from shrimp-originated *Vibrio* spp. in China [135,147,148]. Strains from the VAM and VMB families shared 67–70% amino acid sequence similarities, which suggests their location at two branches which diverged from the same node in the phylogenetic tree [135]. More recently, another novel MBL, Vmh, has been found in *V. alginolyticus, V. chnikoovii, V. mimicus, V. parahaemolyticus, V. fluvialis,* and *V. vulnificus* [71,73]. The amino acid sequence, secondary structure, kinetics assay, and β-lactamase activity have been elucidated by Lu, Hsu, and Lin [73]. The unique sigmoidal hydrolytic curve observed for carbapenem degradation may suggest the substrate-induced dimerization or the cooperative activity of monomeric Vmh [73]. Further examination is necessary to decipher these novel genes’ protein dynamics and conformational heterogenicity.

### 5.3. Resistance Gene Transfer

The increasing prevalence and variety of carbapenemase gene distribution have been evident amongst Gram-negative species in the past decade [9,178]. Once dominantly presented in Enterobacteriaceae, this resistance trait is starting to prevail in once-upon-a-time non-fermenters, including *Vibrio* spp. [9,149]. Genetic analysis indicates that many ARGs identified in the *Vibrio* spp. originate from Enterobacteriaceae [168]. The resistance genes can either be chromosomally encoded or embedded in highly dynamic MGEs such as plasmids and transposons [59,92]. In other words, strains devoid of resistance can acquire the trait by acquiring genes encoding the carbapenemase via HGT (passage of genetic material to non-offspring strain). A single event of HGT can allow the acquisition of multiple fitness factors to promote adaptation to selection pressure [116].

In contrast, the emergence of spontaneous resistance through mutation and vertical transmission (inheritance of heritable genetic material from the parental strain) is relatively slow to sustain evolution. Comprehensive genome analysis confirmed the significance of HGT in propagating the multidrug-resistance and extensively drug-resistant *Vibrio* spp. [59,71,116]. Indeed, members of the Vibrionaceae family manifest remarkable competency in up taking and chromosomally integrating DNA from exogenous sources (see Section 3.1). This could explain the higher HGT rate in Vibrionaceae compared to the other prokaryotes [168]. Substantial MGEs subjected to HGT have been identified in all sequenced *Vibrio* species. They represent more than 10% of the total open reading frames (ORFs) [97].

HGT is a significant factor underlying the rapid dissemination of carbapenem resistance inter- and intraspecies. HGT can be mediated by transduction, transformation, and conjugation [59,116,168]. Chitin is a natural inducer for competence [71,190]. With its primary niche in the aquatic environment and abundance of chitin (animal exoskeleton), *V. cholerae* is a classically outstanding example of competent bacteria [190]. Chitin has been proven to provide a substratum for *V. vulnificus* for attachment and genetic information exchanges [191]. However, the presence of chitin has to be accompanied by the other prerequisites, such as sufficient intracellular cAMP level to facilitate chitin colonization and competence gene expression. Blokesch [190] further demonstrated the association of HapR (the main regulator of quorum sensing) with the transformation and natural competence of *V. cholerae*.

The conjugation efficiency and stability index varied with the recipient species. Interestingly, all the recipient stains recorded significantly higher MIC for imipenem than the parental stain *V. alginolyticus* following the conjugation of a plasmid carrying VMB-1 [147]. This result corroborates with that reported by Zheng, Ye, Chan, and Chen [145] and Cheng, Zheng, Ye, and Chen [135]. Furthermore, convincing findings attested to carbapenemase genes’ stable and transferable expression in *V. cholerae, V. alginolyticus,* and *V. fluvialis* [57,147,149]. Moreover, the transference can occur without antibiotic selection pressure [57]. Stemming from these findings, it is indisputable that *Vibrio* spp. could be the vector disseminating the resistance genes.

Among the myriad MGEs, plasmid is the most commonly identified vehicle for propagating carbapenem resistance among *Vibrio* spp. [192]. Plasmids are extrachromosomal, small circular DNA molecules capable of replicating autonomously [193]. Following the plasmid curing assay, the alteration from imipenem-resistant to susceptible phenotype attested to the plasmid-mediation of carbapenem resistance [27]. Plasmid profiling conducted for *V. parahaemolyticus* sampled from seafood in a study by Venggadasamy, Tan, Law, Ser, Letchumanan, and Pusparajah [32] revealed that 42% of the isolates harbored at least one plasmid, with the highest record at four plasmids. In contrast, 14 different plasmid patterns have been identified during the screening process. The diversity of plasmids is immense, with differing incompatibility types and sizes ranging from 1 kb to hundreds of kb [97]. Plasmids can be classified by the sequences regulating replication through PCR-based replicon typing, a standard identification method. Plasmids sharing the same control systems (replicons) are incompatible and cannot be propagated stably within the same cell line [194].

The size and location of the resistance gene can further be assessed through DNA linearization using S1 nuclease, PFGE, and Southern hybridization [145,147,160]. Plasmid curing assay using ethidium bromide, sodium dodecyl sulphate, acridine orange, and a physical agent offers a more cost-effective and rapid method to cure bacterial plasmids and decipher antibiotic resistance mediation. The resistance is classified as ‘plasmodial’ if affected by the curing and assorted as ‘chromosomal’ if vice versa [195]. The varying prevalence of the different carbapenemase gene variants is surmised to be associated with the location of these genes [175]. The plasmid-mediated resistance is one major cause of AMR [194]. The positive correlation between AMR and plasmid DNA content was reported by Baker-Austin, McArthur, Lindell, Wright, Tuckfield, Gooch, Warner, Oliver, and Stepanauskas [33]. Since the locus of the resistance gene is a determinant for the risk of interspecies dispersion, localizing these genes will provide a more precise conception of the transmission potential rather than simply analyzing the carbapenem resistance status of the strain under investigation.

While some plasmids remain non-typeable [149], carbapenemase genes are mostly plasmid-encoded and often associated with typeable families such as IncA/C, IncC, IncF, IncHI1, IncL/M, and IncN [194,196]. IncA/C demonstrates a broad host range from humans to animals, including aquatic organisms [194,197]. IncC conjugative plasmids are identified as the common propagators of MDR in Vibrionaceae and Enterobacteriaceae. A study revealed that IncC was equipped with the capacity to evade the inhibitory effect from the CRISPR-Cas restriction-modification system of *V. cholerae* to support the adaptation of the plasmid within the strain [168,198]. IncI and IncN have been associated with remarkably efficient conjugative systems. Furthermore, co-resident plasmids’ capacity to be mobilized promotes their propagation and persistence in the host independent of the environmental conditions [194]. The association of the carbapenemase genes with these plasmids implies a high risk of resistance trait dissemination. The dissemination of the promiscuous plasmids overrides the species boundary. Accumulating data reflect the extensiveness of the dissemination of taxonomically diverse pathogens. Notably, very high plasmid similarities have been detected between those identified in *V. cholerae* and those in *Enterobacter aerogenes*, *Photobacterium damselae*, *E. coli,* and *K. pneumoniae* [150,197]. ‘Successful’ plasmids are usually self-conjugative, large (>50kb), and capable of controlling their copy number and regulating their replication rate [194]. However, the involvement of multicopy plasmids in AMR acceleration is often overlooked. Further work is warranted to decipher their role as the evolutionary catalyst for AMR and clonal divergence [98,168].

Gene dissemination via conjugation is likely dependent on several determining factors: MGE stability, gene distance from the origin, conjugative efficiency, host range, populational density, cell physiology, environmental temperature, nutrient availability, humidity, and pH [149,168,194]. Some of the plasmids acquired may be unstable, and the strain can lose the plasmid in subsequent passages. This phenomenon is especially prevalent among non-fermenters. Walsh, Weeks, Livermore, and Toleman [149] concluded that conjugative transfer occurred more efficiently at 30 °C compared to 25 °C or 37 °C. Similarly, the unsuccessful conjugation attempt at 37 °C by Aberkane, Compain, Barraud, Ouédraogo, Bouzinbi, Vittecoq, Jean-Pierre, Decré, and Godreuil [163] reinforced the notion that most of the conjugation took place in an environmental reservoir rather than in the human flora. However, evidence supports the gut microbiome as the provenance of AMR in *V. cholerae* [168]. It was inferred that this thermo-sensitiveness stemmed from the temperature-dependent transcription of a principal gene in the transferred locus, with substantially suppressed expression beyond the optimal temperature [149].

Besides plasmids, transposons are also the primary means of facilitating carbapenem resistance transmission [168,178,199]. For example, *bla*_VMB-2_, a novel MBL that confer resistance to meropenem and cephalosporins, was found on a plasmid-borne composite transposon ISS*hfr*9 in *V. diabolicus* isolated from shrimp sample [148], while the *bla*_VIM-1_ gene identified in *V. alginolyticus* recovered from retail shrimp sample was found associated with a Tn402-like transposon [145]. PCR results demonstrate that VMB-2 gene dissemination by the transposon occurs through the generation of a circular intermediate via a copy-out mechanism that facilitates the transference of the gene to other genomic loci [148]. Analysis of the adjoining genes to the *bla*_NDM-1_ suggests that bearing specific insertion sequences and transposases at the upstream increases the mobility of the resistance gene [57].

The antibiotic resistance genes are also often captured and mediated by integrons [4,133,168]. Integron is an assembly platform that captures ORFs embedded in exogenous gene cassettes via site-specific recombination. It then converts them into functional genes by providing the necessary transcription apparatus [92,139,200,201,202]. Superintegrons are embedded in the chromosome (see Section 3.1); mobile integrons can be incorporated into plasmids or transposons and thus can be laterally mobilized between species [168,203]. Integrons can be classified into five classes based on their integrase sequences [192,200]. Hitherto, classes 1, 2, and 4 have been discovered in *V. cholerae* [204]. Class 1 integron has the highest prevalence in Vibrionaceae isolates and is increasingly recognized as a primary cause of AMR crisis spread, particularly through the Gram-negative rods [133,203]. For example, *bla*_VIM-1_ and *bla*_VIM-4_ were identified within the class 1 integron embedded in a plasmid carried by *V. alginolyticus* and *V. cholerae* [145,163]. It was postulated that multiple resistance genes within the same integron enhance the plasticity in cassette arrangement under antibiotic selective pressure [163]. The integron system enables the reshuffling of genes to encode different virulence functions and multiple antibiotic and heavy metal resistance to enhance adaptation [168,197]. This phenomenon confers a selective advantage to the recipient bacteria and constitutes a major MDR development cause. Most NDM-encoding plasmids co-harbor other resistance genes, such as those encoding for various ß-lactamases, chloramphenicol acetyltransferase, rifampicin ribosyltransferase, and sulfonamide-resistant dihydropteroate synthase [205]. Various antibiotic resistance gene cassettes have been identified as associated with integrons in *V. cholerae* [204,206,207]. These accumulated findings reflect carbapenem resistance’s extensiveness in promoting *Vibrio* spp. through HGT.

### 5.4. Antimicrobial Tolerance

In contrast to antimicrobial resistance development (which resists growth in the presence of an antimicrobial agent), antimicrobial tolerance renders the bacteria the ability to resist killing for an extended period in the presence of a bactericidal agent [169]. Antibiotic tolerance remains an understudied arena. The current research focus has been directed to studying persister cells. Dissimilar to the resistant cells that thrive in the presence of antibiotics, persister cells exist in a dormancy phase. Persister cells are a minor subpopulation variant of dormant cells that are turned into multi-drug resistant strains without undergoing any genetic alteration [93,208,209]. This characteristic can be ubiquitously found in almost all bacteria species [93], including *Vibrio* spp. Although carbapenem resistance is not specified, persister cells have been identified in *V. splendidus* [93], *V. cholerae* in the aquatic body [210], and *V. vulnificus* in the human serum [211]. The dormancy depth depends on multiple factors, including physiological and phenotypical characteristics, protein content, and ribosomal activity. The actual cause for the formation of persister cells remains speculative and could be associated with environmental stress, biofilm microenvironment, social interaction (quorum sensing), or host–pathogen interaction. A controversial inference also exists that the persister cells could arise arbitrarily in an unstressed condition [93]. Among the factors, biofilm formation is the most established and studied phenomenon [212]. A recent report by Sadat, El-Sherbiny, Zakaria, Ramadan, and Awad [75] revealed the remarkable biofilm-forming capability in *V. alginolyticus* (74%) and *V. parahaemolyticus* (76%). Biofilms are aggregates of bacteria encased in a self-produced matrix that protects the assemblage. The biofilm is akin to a shield that limits antibiotic penetration. The ramification of sub-inhibitory concentration exposure is the increment of resilience magnitude to the antimicrobial agent, which made biofilm a formidable reservoir for MDR.

Moreover, some bacteria can activate signaling pathways through quorum sensing and trigger the autolysis of a proportion of cells within the biofilm to release extracellular DNA to sustain the bacteria in the presence of antibiotics [116,213]. Although this adaptive strategy may not render absolute resistance, it promotes tolerance and buys time for continual survival before acquiring other resistance traits [116]. Hence, eradicating biofilm-induced infection is a challenge for nosocomial infections that results in a massive healthcare burden worldwide [214,215].

From another perspective, the ability to assume the spherical morphotype lends impetus to *Vibrio* spp. in developing antimicrobial tolerance. When exposed to a cell wall synthesis inhibitor, the bacteria take the form of a viable but non-dividing spheroplast to protect the cell wall. Upon restoring the permissive condition, the cell initiates division and resumes the typical rod-shaped morphotype by mediating a series of complex stress-sensing systems [169,216,217,218,219]. Espinosa et al. [220] utilized L-arabinose at a low concentration, only 0.01% (w/v), to induce an easily tractable model to study the spherical morphotype. Results suggest that this transient cell-wall deficient state is part of the general physiological response of *Vibrio* sp. to minimize physiological perturbations when confronted by harmful stimuli. This capability has also previously been demonstrated in Gram-negative species such as *P. aeruginosa* [221]. Cross, Ransegnola, Shin, Weaver, Fauntleroy, VanNieuwenhze, Westblade, and Dörr [169] worked out a method to quantify the antimicrobial tolerance based on the OD_600_ measurement (initial and 6 h post-treatment) and colony-forming unit (CFU) count. This phenomenon may affect treatment outcomes as the strain may readily acquire pathogenicity following the cessation of antimicrobial treatment. Worryingly, there were case reports for morphological conversion in clinical specimens post-β-lactam treatment [222,223,224]. In this light, determining the antimicrobial tolerance could also be cataloged as another discriminative test criterion (see Section 4) when studying carbapenem resistance occurrence.

## 6. Dissemination Pathways

**Figure 2:**
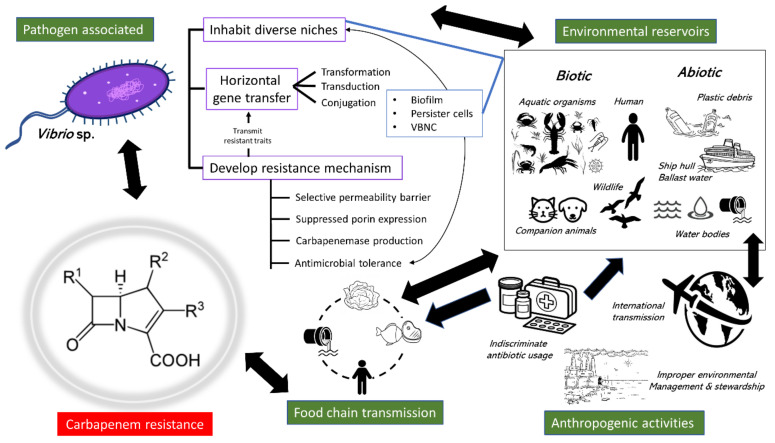
The vicious cycle of carbapenem resistance dissemination.

Before this, carbapenem resistance had rarely been associated with *Vibrio* spp. However, after putting together the discontinuous and sporadic surveillance data over the years, acquired at various regions around the globe (see Section 4), an apparent increasing trend of carbapenem-resistant *Vibrio* spp. is witnessed. Most importantly, non-conventional screening approaches such as genotypic screening for carbapenemase genes revealed that susceptible *Vibrio* strains identified through AST could be carriers for carbapenemase genes and are silently circulating the resistant traits. The mounting evidence is indeed worrying. Moreover, the cause of occurrence is complex, intertwining multiple factors (see Figure 2). Resistance to carbapenem is a function of several biotic and abiotic factors, including pathogen-associated, anthropogenic activity, and the environmental factors. In light of this, attaining a comprehensive overview of the routes of carbapenem resistance dissemination is of paramount importance, given the significance of the healthcare impact from a global perspective. Filling this gap would effectively identify the dissemination fronts and allow expedient initiatives tailored to suppressing the resistance to be dispatched.

### 6.1. Excessive Clinical Usage

AMR emergence has always been directly associated with the indiscriminate usage of antimicrobial agents in clinical settings. The evolutionary adaptation mechanism developed in the pathogen to counteract the offensive effect of antibiotics and to safeguard species continuation is inevitably a natural process sculpted by the selective pressure exerted under the impact of widespread antibiotics usage. There is also evidence for cross-antibiotic resistance. For instance, fluoroquinolone exposure was positively correlated with carbapenem-resistant strains in *A. baumannii*, probably attributed to the activation of intrinsic resistant mechanisms such as the drug efflux pump function [225]. Resistance development tailing the inception of antibiotic application is indeed a foreseeable sequel, and it is no exception for the case of last-line agents such as carbapenems.

Moreover, carbapenem usage is accelerated due to the increasing prevalence of ESBL producers. This became a dominant driving force propelling carbapenem resistance and skewed the selection for carbapenemase-producing strains [58,176]. For instance, in India there was a significant increase in carbapenem resistance post-2011 due to the drastic rise in carbapenem usage since 2010 [226]. A similar trend could also be expected in countries with high carbapenem retail sales, such as Pakistan and Egypt [227,228]. However, the variation in the extent of resistance development is dependable on the stringency of antibiotic usage regulation, the effectiveness of infection control measures, and the efficacy of containing the resistance in the region [47].

Hospitals are potentially the main reservoirs for carbapenem resistance [229]. Patients at risk of acquiring carbapenem-resistant strains may include those with a history of antibiotic usage, long-term hospital admission from endemic areas, close contact with another colonized patient, elderly, immunocompromised, and those with medical complications or poor health status [3,55,57]. Hospital runoff could be regarded as the main point of discharging the carbapenem-resistant strains into the environment [230]. The exposure to carbapenem enables the resistant bacteria to thrive in the effluent without competition from susceptible bacteria. To illustrate, Al Salah, Ngweme, Laffite, Otamonga, Mulaji, and Poté [230] employed both molecular and cultivable approaches to quantify the contribution of hospital effluent in propagating antimicrobial resistant bacteria (ARBs) and ARGs by studying rivers with hospitals as the sole contamination source. Their findings demonstrated a significantly higher prevalence of carbapenem-resistant Enterobacteriaceae and *E. coli* at the hospital sewage discharge point and downstream regions, which is not observed in the upstream areas. The rate of occurrence of *bla*_IMP_ was lowest at the upstream. The prevalence of *bla*_IMP_ at the hospital sampling site was found to be 78-fold higher than at the upstream sampling site in the wet season and 4.5-fold higher in the dry season [230]. Similarly, Lamba et al. [231] reported significantly high levels of CRE and *bla*_NDM-1_ in the urban hospital wastewater, which can be up to nine-fold higher than in local sewages in India. This also corroborates the fact that identifying similar plasmid backbones between patient-hosted and environmental carbapenemase-producing organisms (CPOs) is another cause of concern [232]. This is in line with the Enterobacterial Repetitive Intergenic Consensus polymerase chain reaction (ERIC-PCR) and pulsed-field gel electrophoresis (PFGE) results that showed high similarities between *V. parahaemolyticus* strains in clinical and environmental isolates [34]. The influx of improperly treated hospital sewage into other water bodies thus becomes the main driver for resistance spread. It expedites the dissemination of resistant strains to the immediate environment [233,234]. Evidence regarding the escalating dissemination of hospital-associated infection into community settings has created a significant dilemma for clinicians [3].

Although AMR is conventionally associated with indiscriminate antibiotic usage, some reports contradict the notion that the frequency of antibiotic resistance always correlates with antibiotic exposure [33]. Notably, passive resistance which arises from the direct selection pressure conferred by the application of a particular antibiotic and passive resistance referring to general adaptive measures not associated with any antibiotic are also possible. For instance, *V. fluvialis* was surmised to acquire the *bla*_NDM-1_ gene in the absence of antibiotic selective pressure in a recent finding. However, the strain was reported to be able to transfer this gene to the gut microbial population [57]. Similarly, Baker-Austin, McArthur, Lindell, Wright, Tuckfield, Gooch, Warner, Oliver, and Stepanauskas [33] discovered the formation of a multi-drug resistant *V. vulnificus* environmental isolate which is independent of the clinical reservoir. This implies that there may be other factors contributing to carbapenem resistance.

### 6.2. Transmission via the Food Chain

Besides the clinical application, antimicrobial agents are widely applied in agriculture and aquaculture to control infectious diseases and enhance production. Excessive antibiotic usage gradually alters pathogens’ transmission pattern, virulence profile, and distribution pattern [119,235]. A recent genomic analysis attests to the direct association of resistant mobile genetic elements in *V. parahaemolyticus* with antibiotic usage in shrimp farms. In a recent genomic analysis, Fu et al. [236] demonstrated the direct association of resistant mobile genetic elements in *V. parahaemolyticus* with antibiotic usage. *Vibrio* spp., in particular, *V. parahaemolyticus,* is a common infectious agent causing acute hepatopancreatic necrosis disease (AHPND) in shrimp which often causes mass mortality of shrimp and results in massive economic losses [236]. Although carbapenem is not the drug of choice and has never been licensed for aquaculture application, carbapenem-resistant *V. parahaemolyticus* has been identified in aquaculture farms in India [34]. Moreover, *Vibrio* spp. isolated from the water and sediment samples of shrimp farms are found to produce carbapenemase (4%) [34] and carry the *bla*_NDM-1_ gene (14%) [151]. The results imply that farms can be reservoirs for clinically relevant resistance without direct selection pressure from carbapenem application. This effect could be mediated by HGT or effluent runoff from contaminated sources [124]. Equally concerning are the agricultural fields where the manure, slurry, and bedding materials can act as a source for antibiotic resistance mediated through the soil ecosystem [237].

These resistance traits can be taken up by livestock or crops and transmitted down the food chain to human hosts. With the fecal–oral route widely accepted as a standard mode for *V. cholerae* transmission, it is rational to speculate on the underlying risk of acquiring carbapenem-resistant traits through consuming contaminated water or food. Undercooked seafood is one of the most prevalent sources of microbial contamination [27,30,32,75,133,134]. Contamination may occur during various stages, such as farming, harvesting, processing, packaging, transportation, and storage [121]. Not surprisingly, dietary patterns attributed to geographical variations have been associated with infection trends [72]. The risk is exceptionally high but not confined to countries with high seafood produce. With the advances in the transport chain, contaminated seafood originating from carbapenem-resistant endemic regions can be consumed by at-risk individuals in different parts of the globe [71]. For instance, a *V. cholerae* strain carrying VCC-1- containing plasmid and resistant to IMI, MER, ETP, and DOR was identified in shrimp imported from India to Canada [31]. Imported food could be a concealed source for spreading resistance that is too often overlooked in surveillance studies.

Besides food-producing animals, plant-based food is also increasingly recognized as a potential vehicle for the transference of antimicrobial-resistant bacteria. It was alarming when Igbinosa, Beshiru, Igbinosa, Ogofure, and Uwhuba [132] isolated imipenem-resistant *V. parahaemolyticus* strains from an African salad sampled in southern Nigeria. Many vegetables and fruits often become an underestimated threat as they are typically minimally processed or consumed raw, hence retaining more pathogens than those seafood and meat subjected to the protective cooking steps. Contamination can presumably occur when the plant is exposed to the external environment or the food handler [238]. This symbolizes an often-neglected source of contamination that has rarely been captured by surveillance programs [26,238]. Since these food-borne pathogens intersect with the food chain, they can readily transfer the carbapenem resistance trait to human consumers. A global perspective of the ecology of AMR determinants that postulates the interdependence of the environment, animals, and food with human health is warranted [91].

### 6.3. Environmental Reservoirs

With *Vibrio* spp. being an integral part of the natural inhabitants of many aquatic environments, their contribution to the AMR crisis is largely indubitable [59,168]. *Vibrio* spp. thrives in warmer water (>18 °C) with lower salinities (<25 ppt) and lower nitrogen and phosphorus concentrations [20,239,240]. Various aquatic bodies have become a hub supporting HCT occurrence of *Vibrio* spp. This is especially true when they serve as the discharge point of wastewater treatment plants and typical sink for agriculture runoff [157,167,241,242,243]. For example, urban tropical estuaries [20], recreational beaches [60], and coastal waters [130] have been identified as unappreciated reservoirs harboring carbapenem-resistant *Vibrio* strains. The issue is especially problematic in developing countries where the population density is high, the sanitation and sewage systems are inadequate, or the antibiotic stewardship program is less stringent [20,176,244]. The data gathered from public water supply surveillance in India indicate a high association between carbapenem resistance with environmental exposure [149]. PFGE results from another study revealed high similarities between *V. cholerae* strains recovered from patients and the environmental strains [140]. Mounting evidence indicates that carbapenem resistance can spread from the environment and animals to humans and vice versa [100,186]. Islam, Zaman, Islam, Ahmed, and Clemens [100] proffered the human host (chronic carriers of the bacterium), perpetual transmission among humans, animals, and the environment as the four conceptual reservoirs sustaining the inter-epidemic persistence of *V. cholerae*.

The abundance of *Vibrio* spp. is subjected to seasonal changes and salinity fluctuations [99]. Data gathered suggested an apparent climate link with *Vibrio* infections [99,245]. The abundance, pathogenic potential, and frequency of multiple antibiotic resistance in *Vibrio* spp. increased with higher ambiance temperature [20,33]. The higher *Vibrio* abundance during the spring and autumn seasons may be attributed to the rain runoff that increases the nutrient concentrations in the water. Higher nutrient content favors planktonic bloom, which provides a larger chitinous surface for *Vibrio* attachment, thus increasing the abundance of *Vibrio* spp. [99]. Striking evidence demonstrates the association of the global warming phenomenon with the upsurge of vibriosis incidences over the past two decades [71,245,246,247,248]. The warming climate scenario quickens the proliferation of *Vibrio* spp. and increases shellfish-mediated outbreaks and *Vibrio*-associated wound infections in temperate regions and higher latitudes [249,250,251,252]. These early signals should be captured, and better infection preventive strategies should be mapped out in advance. As global climate change is transitioning in favor of the ecology of *Vibrio* spp., cases of Vibriosis are expected to rise in the near future. A higher abundance of *Vibrio* carriers would indirectly exacerbate the transmission of carbapenem resistance genes.

Although *Vibrio* spp.’s proliferation stagnates during colder months, several intrinsic features of *Vibrio* spp. sustain their persistence in the natural reservoirs. Notably, *Vibrio* spp. can enter a dormant phase designated as the viable but non-culturable (VBNC) form to prolong their survival under unfavorable circumstances [99,253]. *Vibrio* spp. in VBNC form loses its flagellum and presents in a smaller spherical shape, which resembles the spore-like stage [245]. Moreover, when existing as a spherical cell, also called the spheroplast form, its cell wall is partially or wholly non-existent, thus ably protecting it from cell-wall targeting antibiotics and making it even more challenging to be eradicated [220,254]. The dormant form can later regain culturability, transmissibility, and infectivity when favorable conditions resume [245,253].

Additionally, adhering to surfaces allows *Vibrio* spp. to attach to biotic and abiotic surfaces. *Vibrio* spp. can reside on the shells of phytoplankton, zooplankton, and crustaceans. Furthermore, *Vibrio* spp. has been associated with higher organisms such as seagulls [163], waterfowl [255], fish [256,257], mussels [258,259], and chironomid egg masses [260], and identified on environmental plastic debris [261,262] and ship hulls [263]. The ability to form biofilm further reinforced their persistence in the environment [264]. The production of polysaccharides regulated by quorum sensing in *Vibrio* spp. aids in immobilizing cells, forming microcolonies, and maturation of biofilm [99]. These survival strategies enable the bacteria to endure stress, scarcity of nutrients, or unfavorable environmental conditions until the next outbreak [99].

The persistence of the carbapenem-resistant *Vibrio* spp. in the environment sustains the circulation of the resistance trait in the ecosystem. A more precise understanding of the role of environmental reservoirs for carbapenem-resistance species would undoubtedly facilitate the implementation of pragmatic strategies in deterring the aggravation of AMR spread (see Section 7).

### 6.4. Anthropogenic Factors

From another perspective, the resultant environmental reservoirs for carbapenem resistance are plausibly a result of various anthropogenic activities. The identified hotspots of AMR are primarily hospital effluent, aquacultural and aquacultural runoff, wastewater treatment discharge, and sewage plants [92]. Additionally, AMR is likely exacerbated by unmonitored tourism and recreational activities in the coastal areas. An unrelenting flow of human traffic invariably results in anthropogenic pollution, which ultimately expedites AMR, enriches ARGs, and shapes the selection of resistant strains in these ecospheres [20,23,60]. Exposure to polluted water sources harboring resistant strains poses a risk of contracting opportunistic infections and acquiring carbapenem resistance [186].

The advent of the globalization era has made international travel more convenient. Although the practicality of transportation has offered better global connectivity, it also serves as a driving force propelling the diffusion of carbapenem resistance in non-endemic regions [3,4]. To illustrate, the emptying and refilling of ballast tank water facilitate the national and international displacement of plankton, protists, viruses, and bacteria in the marine environment [84,139]. Indirectly, cargo ships became propagators of the carbapenem-resistant vectors. Moreover, with the unprecedented high migration rate and convenience of air travel, the resistant clones and bacterial plasmids can be rapidly transported across nations and continents by asymptomatic human carriers. More worryingly, much of this transmission went undetected until it emerged as a source of endogenous infection. In the clinical setting, a UK reference laboratory report showed that more than 50% of the patients carrying the NDM-1 expressing Enterobacteriaceae had recent travel history to India, with the majority being hospitalized in the country [265]. The Indian subcontinent demonstrates a strong epidemiologic association with MBLs [4]. Retrospective studies surmised that NDM-1 has likely been circulating in the subcontinent since 2006 [266]. However, it is difficult to epidemiologically relate the isolates due to the widely divergent proximity of occurrence and isolation dates [57]. More recent reports noted that some of the NDM-1 carriers had no recent history of hospital admission. This might suggest carbapenem resistance could be more than a pure nosocomial challenge. The resistance clones are likely circulating within the community [181,265].

## 7. Future Research Perspectives

Although AMR development is inevitable, the continuous exacerbation of resistance development at a threatening pace is alarming. The glimpses rendered by the sporadic healthcare-associated case reports of carbapenem resistance approximately a decade ago have become widespread globally [4,205]. It is tempting to speculate that AMR will become a formidable challenge that may haunt humanity for decades to come if counteractive measures are not deployed aptly.

Based on the information in Section 6, *Vibrio* spp.’s transmission has evolved from the conventionally recognized oral–fecal linear model to a more complex model of ecological transmission. Accumulated evidence suggests that the dissemination mode of carbapenem-resistance genes associated with Gram-negative isolates is complex and difficult to predict [178]. This is also in line with a recent multivariate analysis’s outcome, which suggests the need for a holistic and multipronged approach to hamper the rapid diffusion of AMR [267].

Primarily, more stringent stewardship should be imposed on carbapenem antibiotics, as excessive unindicated antibiotic usage is one of the main drivers for resistance development. Regulations and proper cascade mechanisms must be implemented to ensure prudent and justified carbapenem usage as a last-line antibiotic. Educating the public to elevate AMR awareness also signifies an effective means as curbing AMR necessitates a united multisectoral approach. Appropriate policies, legislation, and programs can be introduced to foster better public health outcomes [2]. For example, legislation such as the international limit for ballast water screening before discharge can be enforced as part of the resistance control measures [84]. Routine inspection of the discharged effluent can also be imposed. Moreover, screening at-risk populations or travelers with a travel history to endemic regions can be made mandatory to allow earlier identification of carriers for carbapenem-resistant pathogens so that optimized therapy can be continued promptly to break the chain of transmission [176].

Since the spread of ARGs is also a pivotal contributing factor to carbapenem resistance, it is also imperative to address the fundamental issue of hygiene and sanitation, especially in developing countries where *Vibrio* spp. predominates in mitigating water-borne transmission. Often, antibiotic-containing wastewater is directly discharged into the sewage network and the wastewater treatment plants. The risk for antibiotic resistance can be attenuated if the water is treated before the final discharge. Water can be treated using disinfectant chemicals such as chlorine. Chlorine can destroy the microbe’s nucleic acids and cell membranes and, therefore, can be an effective means to inactivate AMR microbes and restrain the spread of ARGs [242]. Chlorination can also be coupled with UV irradiation, ozonation, charcoal, or slow sand filtration to enhance the removal efficiency of ARGs in the wastewater [268,269,270,271]. Quantitative microbiological risk assessment (QMRA) can be employed to evaluate treatment efficiency and water safety [272,273]. Furthermore, novel approaches such as nanotechnology can be used to remove water antibiotics [274,275,276]. The nanoparticles can disinfect antibiotic-resistant bacteria by oxidizing the cellular component, disrupting the cell membrane, damaging mitochondria, and DNA molecules, and interrupting cellular transport [273]. However, the cost and possible pollution issues associated with these techniques must be considered [273,277].

From another perspective, strategizing a coordinated surveillance plan is crucial to prioritize resources for capturing the salient data featuring the transmission profile of carbapenem resistance amongst environmental isolates. It is impossible to screen all the environmental strains to evaluate the extent of carbapenem resistance. In this regard, *Vibrio* species can serve as one of the potential indicator strains attributed to the list of reasons expanded in Section 3. Future studies can evaluate the validity and accuracy of this taxa approach compared to the profile contributed by other resistant determinant strains such as *E. coli*, *Enterococcus* sp., *Campylobacter* sp., and *Salmonella* sp. Furthermore, this genus can serve as an exciting model organism to help strengthen our understanding of the mode and mechanism of resistance transference. Early detection of emerging resistance signs can help guide decision-making and policy planning concerning infection control and resistance containment.

Moreover, monitoring the trend of resistance patterns concerning climatic factors and environmental dynamics can help improve forecasting and formulate a better strategy to confine carbapenem resistance development [159]. Rather than blindly collecting sporadic data, deliberate and systematized sampling would provide more valuable data for modeling purposes. For instance, the distribution of carbapenem resistance *Vibrio* isolates could be presented on a high-resolution nation map by replicating the method used to generate the national *V. cholerae* seroincidence data. The machine-learning model coupled with the Bayesian geostatistical model can be employed to analyze the systematically collected serosurveillance data [278]. Whether these data can be extrapolated to other countries warrants further validation, given the considerable temporal and spatial heterogeneity in the resistance distribution, which is further complicated by the complex factors influencing the evolution, dissemination, and persistence of the ARGs in the ecology [279]. Perhaps the regression model would be a practical means to create a more accurate regional mapping by considering the combined influence of environmental variables [119].

Nevertheless, fostering international collaboration would aid in fabricating a global mapping by combing the incomplete surveillance data gathered in different nations. Currently, the AMR monitoring system is either non-existent or inadequate in many countries [181]. A lack of organized reporting often masks reality and results in underestimated resistance rates, mainly when the initial detection of carbapenem resistance is below the registration threshold [58,280]. A specific surveillance system such as the “Cholera and Other *Vibrio* Illness Surveillance” (COVIS) system and the Canadian Integrated Program for Antimicrobial Resistance Surveillance (CIPARS) established in the United States and Canada can be replicated in other countries [281,282]. Implementing a global surveillance network can help provide critical insights into the propagation pattern of carbapenem resistance [283]. Accurate transmission information is indispensable for devising control measures, particularly in high-risk zones. Investing in preventing and controlling the spread of resistant bacteria can ultimately lower the social and economic cost of AMR [2].

Additionally, the present scope of surveillance is relatively limited, predominantly focused on clinical settings. A more systematic and targeted approach devised by considering the mode of transmission substantiated in the current literature will ensure better resource allocation and prevent data duplication. Previously, environmental surveillance was never subjected to the same level of scrutiny as clinical inspection [31]. With the establishment of environmental *Vibrio* spp. as carriers of carbapenemase and carbapenem-resistant traits, greater attention can be allocated to environmental reservoirs that possibly harbor carbapenem-resistant *Vibrio* spp. Beyond the hospital and agricultural run-offs, wastewater treatment plants and coastal regions with high human visitation rates are also red zones that necessitate routine monitoring. Understanding the selective pressures that sustain the carbapenem-resistance reservoirs in the environment is critical to laying out the proper control measures to slow down the resistance dissemination.

Notably, carbapenem resistance transmission through the food chain has been observed via *Vibrio* spp. isolated from food sources (see Section 6.2). Therefore, routine screening on foodstuffs, particularly raw meat and vegetables, for carbapenem-resistant isolate is urgently needed, although it is not legally required. Furthermore, data on the presence of carbapenem pathogens in animals are also inordinately limited. The magnitude at which animals contribute to the transmission of carbapenem resistance is yet unknown. Identifying the carbapenemase gene in *V. cholerae* of avian origin underscores the importance of screening animal vectors. Convincing data indicated that highly plastic plasmid-mediated resistance is increasingly widespread in wildlife [237].

Similarly, companion animals could also be a source of carbapenem resistance [284]. In this context, the surveillance program can extend to companion animals and wildlife, which could be another neglected reservoir for carbapenem resistance. Apart from that, environmental plastic is seemingly an overlooked source of contamination that aggravates carbapenem resistance. Idle plastic debris in the environment can serve as stable habitats for microorganisms. *Vibrio* spp., for instance, has been identified in high abundance on microplastics as they can ably colonize plastic material through biofilm formation [261,262]. These bacteria could actively receive and disperse the carbapenem resistance genes, thus being a risk to the other organisms sharing the same habitat. There is a pressing need to extend the surveillance beyond the conventionally identified mediums of carbapenem transmission.

Undoubtedly, AST can be an essential tool for resistance surveillance, but this approach may be more labor intensive and generally less sensitive [3,157]. The collaborative effort must be invested in developing innovative diagnostic tools to facilitate more cost-effective, rapid, robust, and continuous worldwide surveillance. Molecular methods such as whole-genome sequencing have become the cornerstone for rapid detection and tracking of resistance carriers [242]. Resistance markers associated with indicator strains such as *Vibrio* spp. can also be pre-identified to ease molecular screening [168]. Moreover, the immunofluorescence and PCR methods can help identify the resistant bacteria in the environmental reservoir during the inter-epidemic period, even in the non-culturable form [100,253]. Furthermore, there are also opportunities to implement remote-sensing-based approaches to monitor environmental matrices [285,286]. These cutting-edge technologies could provide incredible insights into the resistome, infer the evolutionary timeline, and trace the outbreak’s source [116,242]. This information can help formulate proper mitigation strategies to tackle the challenge of resistance transmission.

Last but not least, it is also necessary to reinvigorate the research and development of novel antimicrobial interventions or their substitutes. The new antimicrobial agent must be added to the pipeline, especially those targeting the critically resistant Gram-negative pathogens. Some promising avenues include synergistically combining antibiotics [287] and novel peptides [288], improvising and diversifying carbapenem structure [42], and introducing carbapenemase inhibitors [289,290,291,292,293]. Current research focus seems to prioritize probiotic and synbiotic research, in which bacteria strains secreting antimicrobial peptides and bacteriocins are harnessed as antibiotic alternatives to treat pathogens, thereby helping to control AMR [294,295,296,297,298,299]. There is also renewed interest in the practice of phage therapy [300,301,302] and the discovery of quorum sensing inhibitors [303,304] as alternative therapeutic options for antibiotics. Introducing novel antibiotic alternatives can minimize antibiotic usage and ultimately slow down the rate of carbapenem resistance development.

## 8. Conclusions

Carbapenem resistance is an apparent emerging threat and a hazard from the global perspective. This antibiotic serves as a last-line antimicrobial agent in managing clinical infections. Moreover, there are limited therapeutic options in the pipeline. More alarmingly, this threat is often underestimated due to the lack of surveillance and low public awareness. Based on the discussion and findings above, multiple factors contribute to the driving force of carbapenem resistance transmission in the environment (Figure 2). Carbapenem resistance development is further accelerated through various human activities such as excessive antibiotic usage, improper antimicrobial stewardship, and international travel.

Moreover, propagation of carbapenem resistance is expedited by the expanding pool of bacterial vectors, which acquire the resistance traits primarily through horizontal gene transfer. The transmission of carbapenem resistance involves a complex multi-factorial model. Our writing provided new insights into the extensiveness of carbapenem resistance dissemination among *Vibrio* spp. in the environment. The status report of the distribution of carbapenem-resistant *Vibrio* spp. presented in this review revealed the widespread prevalence of carbapenem-resistant *Vibrio* spp. globally, including in non-endemic regions. We hope that by introducing *Vibrio* spp. as one of the prospectus indicator strains, we can develop a more straightforward, rapid, and systematized way for future monitoring of carbapenem resistance. Furthermore, this current work also contributes to a better knowledge of the mechanisms and modes modulating carbapenem resistance. Identifying the causal pathways of carbapenem transmission is imperative for adequately designing practical control measures and the timely introduction of novel therapeutic options. 

## Figures and Tables

**Table 1:** Prevalence data for carbapenem-resistant *Vibrio* sp. worldwide.

Continent	Country	Sample Type	Vibrio Species (Number of Strain)	Susceptibility to Carbapenem Antibiotics	Carbapenem Resistance Number of Strain (%)			MIC	Carbapenemase Gene Identified (Location)	Year of Isolation	Reference
					R	I	S				
Asia	Bangladesh Chhatak (north-eastern Bangladesh)	Clinical	*V. cholerae O1* (141)	IMI	0	0	141 (100%)	-	Absent	2009–2014	[138]
		Environmental	*V. cholerae O1* (21)	IMI	0	0	21 (100%)	-	Absent		
	Bangladesh Mathbaria (south-western Bangladesh)	Clinical	*V. cholerae O1* (178)	IMI	0	0	178 (100%)	-	Absent		
		Environmental	*V. cholerae O1* (120)	IMI	0	0	120 (100%)	-	Absent		
	Bangladesh Chhatak (north-eastern Bangladesh)	Clinical	*V. cholerae O1* (68)	IMI	0	1 (1%)	67 (99%)	-	-	2013	[139]
		Environmental	*V. cholerae O1* (6)	IMI	1 (17%)	0	5 (83%)	-	-		
	Bangladesh Mathbaria (south-western Bangladesh)	Clinical [diarrheal sample]	*V. cholerae O1* (77)	IMI	0	0	77 (100%)	-	-		
		Environmental [ponds]	*V. cholerae O1* (57)	IMI	0	0	57 (100%)	-	-		
	China [16 counties]	Clinical (429) Environment [water (77), soil/surface (62)]	*V. cholerae* El Tor (568)	IMI	568 (100%)	0	0	-	-	1986–2012	[140]
				MER	57 (10%)	0	0	-			
	China (Mohnarin hospitals in different areas)	Clinical	*V. parahaemolyticus (51)*	Carbapenem	-	-	(>90%)	-	-	2010	[141]
	China (south-eastern China)	Clinical [diarrheal sample]	*V. parahaemolyticus (563)*	Carbapenem	-	-	(>95%)	-	-	2015	[142]
	China [6 counties]	Clinical [diarrheal sample]	*V. parahaemolyticus (2871)*	IMI	(<1%)	-	-	Interpretive standard of resistance: MIC ≥ 4 µg/mL	-	2016–2020	[118]
	China Zhejiang	Freshwater farm, Seawater farm, Market	*V. parahaemolyticus (360)*	IMI (10 µg)	-	-	360 (100%)	-	-	2017–2019	[143]
	China	Seafood [shrimp]	*V. alginolyticus* Vb1394	IMI	0	1	0	2 mg/L	-	2018	[144]
				MER	0	1	0	2 mg/L	-		
	China Shenzhen	Seafood [shrimp]	*V. alginolyticus* Vb1978	IMI	-	-	-	0.5 mg/L	NDM-1, VIM-1 (plasmid;50 kb)	2019	[145]
				MER	-	-	-	0.03 mg/L			
	China Bohai Bay, Tianjin	Environmental [Coastal seawater)]	*Vibrio* sp. (carrying *bla_OXA58_*)	IMI	-	-	-	2 µg/mL	OXA-58 (plasmid)	2019	[23]
				MER	-	-	-	8 µg/mL			
	China	Clinical [diarrheal sample] Seafood [from markets]	*V. parahaemolyticus (107)*	Carbapenem	-	-	(90–100%)	-	-	2019	[146]
	China	Seafood [shrimp]	*V. alginolyticus* Vb1796	IMI	-	-	-	0.12 mg/L	VMB-1	2020	[147]
				MER	-	-	-	1 mg/L			
	China	Seafood [shrimp]	*V. alginolyticusVb1579*	IMI	0	0	1	Standard agar dilution method: (0.06 mg/L)	VAM-1	2021	[135]
				MER	0	0	1	(0.12 mg/L)			
	China	Seafood [shrimp]	*V. diabolicus* *SLV18*	IMI	0	0	1	-	VMB-2 -	-	[148]
				MER	-	-	-	4 µg/mL (Highly resistant)			
	China	collection center	*V. vulnificus*	-	-	-	-	-	Vmh (chromosome)	-	[73]
	India Kolkata (east India) Delhi (north India)	Clinical [diarrheal sample]	*V. cholerae (443)*	IMI	~ <20 (~ <5%)	-	-	-	NDM-1 (chromosome)	2008–2015	[59]
	India	Clinical [diarrheal sample]	*V. fluvialis (115)*	IMI	27 (24%)	-	-	4-32 µg/mL	27 (24%) NDM-1 (Class 1 integron) 27 (24%) OXA-1, OXA-7, OXA-9	2009–2013	[57]
	India Central New Delhi	Environmental [seepage, tap water, sewage effluent]	*V. cholerae* 116-14	IMI	-	-	-	8 mg/L	NDM-1 (chromosome and plasmid; 400 kb)	2010	[149]
				MER	-	-	-	8 mg/L			
			*V. cholerae* 116-17	IMI	-	-	-	16 mg/L	NDM-1 (plasmid Inc A/C;170kb)		
				MER	-	-	-	1 mg/L			
	India Puducherry	Clinical [fecal sample of a child]	*V. cholerae* O1 El Tor Ogawa	-	-	-	-	-	NDM-1 (plasmid)	2012	[150]
	India (Southwest coast)	Environmental [water, sediment sample from estuary]	*Vibrio sp. (180)*	-	-	-	-	-	(13%) NDM-1	2012	[151]
		Environmental [water, sediment sample from shrimp farms]	*Vibrio sp. (70)*	-	-	-	-	-	(14%) NDM-1		
		Seafood [retail]	*Vibrio sp. (30)*	-	-	-	-	-	(7%) NDM-1		
	India Kerala	Environmental [sediment, water sample from aquaculture farm] Seafood [shrimp]	*V. parahaemolyticus (27)*	MER (10 µg)	1	8	18	-	(4%) produces carbapenemase	2015–2016	[34]
	Korea	Seafood [10 types]	*V. parahaemolyticus*	IMI	(70%)	(13%)	(17%)	-	-	2009	[134]
			*V. parahaemolyticus*	MER	(50%)	(29%)	(221%)	-	-		
	Korea Southern coast of South Korea	Environment [mud, tidal water]	*V. parahaemolyticus (1720)*	MER (10 µg)	4 (<1%)	-	-	64 µg/mL 128 µg/mL 128 µg/mL 1024 µg/mL	-	2013–2014	[152]
	Korea Cheongju	Seafood [shrimp from retail outlet]	*V. parahaemolyticus (27)*	IMI	-	-	-	MIC_50_ = ≤1 mg/L; MIC_90_ = ≤1 mg/L Imipenem (range = 1–8 mg/L)	-	2016	[153]
	Korea	Seafood [Cockles]	*V. parahaemolyticus (4)*	IMI (10 µg)	0	3 (75%)	1 (25%)	-	-	-	[82]
			*V. alginolyticus (11)*	IMI (10 µg)	0	3 (27%)	8 (73%)	-			
			*V. diabolicus (14)*	IMI (10 µg)	0	5 (36%)	9 (64%)	-			
			*V. harveyi (3)*	IMI (10 µg)	0	2 (67%)	1 (33%)	-			
	Korea	Seafood [hard shell mussel]	*Total Vibrio* sp. *(32)* *V. parahaemolyticus (2)* *V. harveyi (1)* *V. alginolyticus (13)* *V. diabolicus (16)*	IMI (10 µg)	(3%)	(3%)	(94%)	-	1(3%) OXA [*V. diabolicus*]	-	[133]
	Malaysia Selangor	Seafood [freshwater fish]	*V. parahaemolyticus (49)* *V. cholerae (8)*	IMI	0	0	57 (100%)	-	-	-	[154]
	Malaysia Selangor	Seafood [shellfish]	*V. parahaemolyticus (200)*	IMI (10 µg)	1 (<1%)	18 (9%)	181 (90%)	-	-	2014	[29]
	Malaysia	Seafood [shrimp]	*V. parahaemolyticus (185)*	IMI (10 µg)	4 (2%)	-	-	-	-	2014	[30]
		Seafood [shrimp and shellfish]	*V. parahaemolyticus (385)*	IMI (10 µg)	5 (1%)	18 (5%)	362 (94%)	-	-	-	[28]
	Malaysia Selangor	Seafood [marine and freshwater fish]	*V. parahaemolyticus (165)*	IMI (10 µg)	19 (12%)	6 (4%)	140 (85%)	-	-	2016	[27]
	Malaysia Selangor	Seafood [shrimps, clams, squid]	*V. parahaemolyticus (120)*	IMI (10 µg)	0	2 (2%)	118 (98%)	-	-	2018	[123]
				MER (10 µg)	0	2 (2%)	118 (98%)	-			
	Malaysia Selangor	Seafood [shellfish]	*V. parahaemolyticus (43)*	IMI (10 µg)	10 (23%)	0	33 (77%)	-	-	-	[32]
	Singapore	Environment [harbor, ballast water from ships]	*V. alginolyticus*	MER	3	-	-	-	-	2016	[84]
			*V. parahaemolyticus*	MER	2	-	-	-	-		
			*V. vulnificus*	MER	6	-	-	-	-		
			*V. brasiliensis*	MER	1	-	-	-	-		
			*V. campbellii*	MER	18	-	-	-	-		
			*V. rotiferianus*	MER	1	-	-	-	-		
			*V. tubiashii*	MER	1	-	-	-	-		
	Vietnam (Southern Vietnam)	Environmental	*V. cholerae (100)* non-O1, non-O139	-	-	-	-	-	3 (3%) NDM-1	2010–2013	[155]
Oceania	Australia Northern territory	Clinical [infection site]	*Vibrio sp. (44)*	MER	-	-	(93%)	-	-	2000–2013	[156]
Africa	Egypt Mansoura	Seafood [fish and shellfish]	*V. parahaemolyticus (50)*	IMI (10 µg)	12 (24%)	4 (8%)	34 (68%)	-	-	-	[75]
			*V. alginolyticus (42)*	IMI (10 µg)	8 (19%)	10 (24%)	24 (57%)	-	-		
	Nigeria (South-south Nigeria)	Seafood [shellfish]	*Vibrio* sp. (6)	IMI (10 µg)	6 (100%)	0	0	-	-	2015–2017	[121]
	Nigeria (Eight states in Southern Nigeria)	African salad	*V. parahaemolyticus (63)*	IMI (10 µg)	2 (3%)	7 (11%)	54 (86%)	-	-	2018–2019	[132]
	Nigeria Lagos	Environment [sea water, wet and dry sand]	*V. parahaemolyticus (26)*	IMI	(4%)	-	-	-	1 (4%) NDM-1	-	[60]
				MER	(4%)	-	-	-	-		
				ETP	(4%)	-	-	-	-		
			*V. vulnificus (14)*	IMI	(57%)	-	-	-	5 (36%) NDM-1		
				MER	(57%)	-	-	-	-		
				ETP	(57%)	-	-	-	-		
	South Africa [Chris Hani, Amahlathi, Lukhanji]	Environment [Final effluents from dams, earth canals, rivers, receiving water bodies, tap water and wastewater treatment units]	*V. cholerae (61)*	IMI (10 µg)	0	2 (3%)	59 (97%)	-	19 (31%) *NDM-1, GES, IMP, VIM* 14 (23%) produce carbapenemase	2018	[157]
				MER (10 µg)	0	0	61 (100%)	-			
				ETP (10 µg)	5 (8%)	3 (5%)	53 (87%)	-			
				DOR (10 µg)	1 (2%)	2 (3%)	59 (97%)	-			
	South Africa [Eastern Cape]	Environment [wastewater effluent]	Total *Vibrio* sp. *(52)* *V. parhaemolyticus (12)* *V. vulnificus (18)* *V. fluvialis (19)* *V. metschnikovii (3)*	IMI (10 µg) MER (10 µg)	0	0	52 (100%)	-	-	2010	[158]
	South Africa [Eastern Cape]	Environment [river water]	Total *Vibrio* sp. (118)	IMI (10 µg)	39 (33%)	40 (34%)	39 (33%)	-	35(30%) VIM, 13 (11%) OXA-48, 18 (15%) IMP, 20 (17%) GES, 7 (6%) KPC	2018	[131]
				MER (10 µg)	32 (27%)	21 (18%)	65 (55%)	-	-		
			*V. mimicus (40)*	IMI (10 µg)	2 (5%)	17 (43%)	21 (53%)	-	-		
				MER (10 µg)	7 (18%)	8 (20%)	25 (63%)	-	-		
			*V. vulnificus (37)*	IMI (10 µg)	26 (70%)	10 (27%)	1 (3%)	-	-		
				MER (10 µg)	17 (46%)	7 (19%)	13 (35%)	-	-		
			*V. fluvialis (41)*	IMI (10 µg)	11 (27%)	13 (32%)	17 (41%)	-	-		
				MER (10 µg)	8 (20%)	6 (15%)	27 (66%)	-	-		
	Uganda (South-western district)	Environmental [surface water]	*Vibrio* sp. (392)	MER (10 µg)	180 (46%)	38 (10%)	174 (44%)	-	-	2019	[159]
Europe	Italy [Northern Sardinia]	Environmental [water sample from coast and gulf]	*V. alginolyticus (40)*	IMI (10 µg)	-	-	-	MIC_50_ = 0.06 mg/L; MIC_90_ = 0.12 mg/L Imipenem (range = 0.06–2 mg/L)	-	-	[85]
				MER (10 µg)	-	-	-	MIC_50_ = 0.06 mg/L; MIC_90_ = 0.06 mg/L Meropenem (range = 0.03–0.25 mg/L)	-		
			*V. parahaemolyticus (8)*	IMI (10 µg)	-	-	-	MIC_50_ = 0.125 mg/L; MIC_90_ = 1 mg/L Imipenem (range = 0.06–1 mg/L)	-		
				MER (10 µg)	-	-	-	MIC_50_ = 0.006 mg/L; MIC_90_ = 0.12mg/L Meropenem (range = 0.004–0.12 mg/L)	-		
			*V. vulnificus (6)*	IMI (10 µg)	-	-	-	MIC_50_ = 0.12 mg/L; MIC_90_ = 0.12 mg/L Imipenem (range = 0.06–2 mg/L)	-		
				MER (10 µg)	-	-	-	MIC_50_ = 0.006 mg/L; MIC_90_ = 0.12mg/L Meropenem (range = 0.004–0.25 mg/L)	-		
	Italy	Environmental [Seawater]	*V. cholerae (12)*	MER (10 µg)	0	6 (50%)	6 (50%)	-	-	2003–2014	[160]
		Environmental [Freshwater]	*V. cholerae (5)*	MER (10 µg)	0	1 (20%)	4 (80%)	-	-		
		Seafood	*V. cholerae (25)*	MER (10 µg)	1 (4%)	4 (16%)	20 (80%)	-	-		
	Italy (north-western Adriatic Sea coasts)	Seafood [European Seabass]	*V. anguillarum* *28AD*	IMI (10 µg)	0	0	1	4 µg/mL	-	2007–2011	[161]
		Environmental [Water from Celeri Lagoon]	*V. parahaemolyticus* *VPE116*	IMI (10 µg)	0	0	1	0.125 µg/mL	-		
	Italy (north-western Adriatic Sea coasts)	Seafood [shellfish] Wildlife [turtle blood] Environmental [beach, brackish water]	*V. vulnificus (40)*	IMI (10 µg)	0	0	40 (100%)	-	-	-	[162]
				MER (10 µg)	0	0	40 (100%)	-			
	France Port-Saint-Louis	Wildlife [gull]	*V. cholerae* non-O1/non-O139	IMI	-	-	-	3 mg/L	VIM-1, VIM-4 (plasmid)	2013	[163]
				MER	-	-	-	0.5 mg/L			
				ETP	-	-	-	0.19 mg/L			
				DOR	-	-	-	0.75 mg/L			
	France (imported)	Seafood [shrimp]	*V. parahaemolyticus*	IMI	0	0	(100%)	-	NDM-1 Produces carbapenemase	2016	[74]
	Germany [Baltic Sea, the North Sea, Ems and Weser River estuaries]	Total (184)	*V. cholerae* *V. vulnificus*	IMI (10 µg)	(2%)	(1%)	(97%)	-	-	2004–2014	[21]
				MER (10 µg)	(<1%)	(2%)	(98%)				
		Retail (35)	*V. cholerae* *V. vulnificus*	IMI (10 µg)	0	0	(100%)	-	-		
				MER (10 µg)	0	0	(100%)				
		Clinical (18)	*V. cholerae* *V. vulnificus*	IMI (10 µg)	0	0	(100%)	-	-		
				MER (10 µg)	0	0	(100%)				
		Environmental (131)	*V. cholerae* *V. vulnificus*	IMI (10 µg)	(3%)	(2%)	(95%)	-	-		
				MER (10 µg)	(1%)	(2%)	(97%)				
		North Sea (52)	*V. cholerae* *V. vulnificus*	IMI (10 µg)	(6%)	0	(94%)	-	-		
				MER (10 µg)	(2%)	(4%)	(94%)				
		Baltic Sea (79)	*V. cholerae* *V. vulnificus*	IMI (10 µg)	(1%)	(3%)	(96%)	-	-		
				MER (10 µg)	0	(1%)	(99%)				
	Germany [Baltic Sea, the North Sea]	Environmental [Coastal water]	*V. cholerae (4)*	IMI MER	4 (100%)	0	0	-	3 VCC-1	-	[130]
	Norway [temperate and Polar Oceanic area]	Environmental [Seawater, sea creatures]	*V. alginolyticus (53)*	IMI (10 µg)	0	2 (4%)	51 (96%)	2–8 µg/mL	-	2018	[83]
				MER (10 µg)	0	0	53 (100%)	-			
			*V. anguillarum (21)*	IMI (10 µg)	21 (100%)	0	0	-	-		
				MER (10 µg)	0	0	21 (100%)	-			
			*V. antiquaries (2)*	IMI (10 µg) MER (10 µg)	0	0	2 (100%)	-	-		
			*V. fujianensis (2)*	IMI (10 µg)	0	1 (50%)	1 (50%)	-	-		
				MER (10 µg)	0	0	2 (100%)	-			
			*V. metschnovikovii (38)*	IMI (10 µg) MER (10 µg)	0	0	38(100%)	-	-		
	Slovakia (Eastern and southern)	Environmental [freshwater]	*V. cholerae (21)*	IMI (10 µg)	0	8 (38%)	13 (62%)	-	-	2016	[70]
	Spain	Clinical [leg ulcer]	*V. metschnikovii*	IMI	0	0	1	<1 µg/mL	-	2008	[164]
	UK	Clinical [blood sample of a burn patient]	*V. cholerae*	MER	1	0	0	-	NDM-1	2011	[165]
South America	Brazil	Seafood [shrimp]	*Vibrio (26)*	IMI (10 µg) [in seawater]	1 (4%) [*V.* *navarrensis*]	0	25 (96%)	-	-	-	[81]
				IMI (10 µg) [in distilled water]	0	0	26 (100%)	-			
	Brazil Guanabara Bay	Environment [water sample from polluted estuary]	*V. parahaemolyticus (150)*	IMI	1 (<1%)	2 (1%)	147 (99%)	-	-	2018–2019	[20]
		Environment [water sample from polluted estuary]	*V. alginolyticus (1)*	IMI	1	0	0	-	-		
	Ecuador Cuenca	Seafood [shrimp]	*V. parahaemolyticus (154)*	IMI (10 µg)	(<1%)	0	(99%)	-	-	2012	[122]
				MER	0	0	(100%)	-	-		
North America	Maryland Coastal Bays, Chesapeake Bay	Environmental [Surface water]	*V. parahaemolyticus (77)*	IMI	0	0	77 (100%)	(IMI; 2–16 µg/mL)	-	2009	[166]
				MER	0	0	77 (100%)	(MERO; 2–16 µg/mL)			
			*V. vulnificus (120)*	IMI (10 µg)	2(2%)	0	118 (98%)	-	-		
				MER (10 µg)	0	0	120 (100%)	-			
	Canada (imported)	Seafood [shrimp; imported from India]	*V. cholerae*	DOR, ETP, IMI, MER	1	0	0	>32 µg/mL	VCC-1 (plasmid)	2014	[31]
-	-	Culture collection center	*V. cholerae (20)*	IMI (10 µg)	-	-	-	MIC_50_ = 0.25 µg/mL; MIC_90_ = 0.25 µg/mL Imipenem (range = 0.0–0.5 µg/mL)	-	-	[51]
				MER (10 µg)	-	-	-	MIC_50_ = 2 mg/L; MIC_90_ = 2 mg/L Meropenem (range = 1–2 mg/L)			
			*V. parahaemolyticus* (20)	IMI (10 µg)	-	-	-	MIC_50_ = 0.06 µg/mL; MIC_90_ = 0.06 µg/mL Imipenem (range = 0.06 µg/mL)	-		
				MER (10 µg)	-	-	-	MIC_50_ = 0.06 mg/L MIC_90_ = 0.5mg/L Meropenem (range = 0.06–0.5 mg/L)			
			*V. vulnificus* (20)	IMI (10 µg)	-	-	-	MIC_50_ = 0.06 µg/mL Imipenem (range = 0.06 µg/mL)	-		
				MER (10 µg)	-	-	-	MIC_50_ = 1.12 mg/L; MIC_90_ = 0.25 mg/L Meropenem (range = 0.06–0.25 mg/L)			

R: resistance, I: intermediately resistant; S: susceptible; IMI: imipenem; MER: meropenem; ETP: ertapenem; DOR: doripenem; GES: Guiana extended-spectrum; IMP: Imipenemase MBL; active-on-imipenem; KPC: *K. pneumoniae* carbapenemase; NDM: New Delhi metallo-ß-lactamase; OXA: Oxacillinases; VAM: *Vibrio alginolyticus* MBL; VIM: Verona integron-encoded MBL; VMB: *Vibrio* MBL; Vmh: MBL fold metallohydrolase.

**Table 2:** Classification of various carbapenemases.

Ambler Class	Active Site	Representative Carbapenemase Type	References
A	Serine	*K. pneumoniae* carbapenemase (KPC) * VCC * Imipenemase MBL (IMP) * Guiana extended-spectrum (GES) * Serratia fonticola carbapenemase (SFC) *Serratia marcescens* enzyme (SME) Non-metallo-carbapenemase-A (NMC-A)	[4,41,47,152]
B	Zinc (cofactor)	New Delhi MBL (NDM)* Imipenemase; active-on-imipenem (IMP) * Verona integron-encoded MBL (VIM) * German imipenemase (GIM) Seoul imipenemase (SIM) Australian imipenemase (AIM-1) Dutch imipenemase (DIM-1) Florence imipenemase (FIM-1) *S. marcescens* MBL (SMB-1) Sao Paulo MBL(SPM) Kyorin Health Science MBL (KHM-1) Tripoli MBL (TMB-1) Pseudomonas Fluorescens MBL (PFM) *Vibrio alginolyticus* MBL (VAM) * Vibrio MBL (VMB) * MBL fold metallohydrolase (Vmh) *	[47,73,147,183]
C	Serine	Not considered as carbapenemase	[47]
D	Serine	Oxacillinases (OXA) * OXA-23 OXA-24/-40 OXA-25 OXA-26 OXA-27 OXA-40 OXA−48 * OXA-49 OXA−51 OXA−58 * OXA-72 OXA-143	[42,149]

* Indicates the carbapenemase (including phenotypic and genotypic) identified in *Vibrio* spp.

## Data Availability

Not applicable.
